# Trends in advanced HIV disease, treatment interruption, and viraemia in KwaZulu-Natal, South Africa

**DOI:** 10.1371/journal.pgph.0005506

**Published:** 2026-09-02

**Authors:** Sanele S. Mbeje, Lilishia Gounder, Johan S. van der Molen, Lungile Hobe, Thulani Ngwenya, Mlungisi Khanyile, Kwena Tlhaku, Benjamin Chimukangara, Thokozani Khubone, Yukteshwar Sookrajh, Sharana Mahomed, Jennifer A. Brown, Nigel Garrett, Jienchi Dorward, Lara Lewis

**Affiliations:** 1 Centre for the AIDS Programme of Research in South Africa (CAPRISA), University of KwaZulu-Natal, Durban, South Africa; 2 Department of Virology, School of Laboratory Medicine and Medical Sciences, University of KwaZulu-Natal, Durban, South Africa; 3 Department of Virology, National Health Laboratory Service, Inkosi Albert Luthuli Central Hospital, Durban, South Africa; 4 Mseleni Hospital, uMkhanyakude, KwaZulu-Natal, South Africa; 5 Bethesda Hospital, uMkhanyakude, KwaZulu-Natal, South Africa; 6 Discipline of Public Health Medicine, School of Nursing and Public Health, University of KwaZulu-Natal, Durban, South Africa; 7 Critical Care Medicine Department, NIH Clinical Center, Bethesda, Maryland, United State of America; 8 eThekwini Municipality Health Unit, Durban, South Africa; 9 Department of Medical Microbiology, University of KwaZulu-Natal, Durban, South Africa; 10 Nuffield Department of Primary Care Health Sciences, University of Oxford, Oxford, United Kingdom; 11 Desmond Tutu HIV Centre, University of Cape Town, Cape Town, South Africa; University of Cape Town Faculty of Health Sciences, SOUTH AFRICA

## Abstract

Advanced HIV disease (AHD), treatment interruption and viraemia remain key challenges to effectiveness of HIV programmes in South Africa. Understanding the trends of these measures and their variation across geographic regions can inform more targeted and responsive interventions. We conducted a retrospective cohort study using routine, de-identified data from TIER.Net, a national HIV electronic register. We curated data from 116 primary care clinics in eThekwini, uMgungudlovu, and uMkhanyakude Districts in KwaZulu-Natal province. We included people living with HIV aged ≥16 years, who initiated or collected ART between 1 July 2018 and 30 June 2023. We examined trends in the annual clinic-level prevalence of AHD, treatment interruption and viraemia, and mapped these outcomes using inverse distance weighted interpolation restricted to 3 nearest neighbours to preserve local influence. Among 123,473 clients initiating ART with CD4 count measurements, the overall prevalence of AHD ranged between 20.0% and 22.2%. The prevalence of treatment interruptions ranged between 11.3% and 13.2% among the 544,066 clients who had scheduled visits. Of the 446,899 clients who had viral load results, overall viraemia, defined as ≥50 copies/mL, increased from 12.9% to 20.2%, whereas high-level viraemia defined as ≥1000 copies/mL remained stable at approximately 6%. Spatial analyses highlighted geographic disparities in AHD, treatment interruption, and viraemia across and within the districts. The persistence of AHD, treatment interruption and viraemia require further investigation and highlight ongoing challenges for HIV control. The observed spatial variation across and within districts spanning urban, peri-urban and rural settings highlight the need for geographically tailored interventions to improve programmatic effectiveness, particularly in hyper-endemic settings.

## Introduction

Human immunodeficiency virus (HIV) continues to be one of the most challenging public health issues in South Africa (SA) and globally. Despite significant advances, SA remains the epicentre of the HIV epidemic [1,2]. The country hosts one of the largest HIV treatment programmes and has made progress in identifying untreated people living with HIV (PLWH), expanding access to testing and care services, and improving viral suppression [1]. However, HIV prevalence is not evenly distributed across regions. The KwaZulu-Natal province has one of the highest HIV prevalences in the country, estimated at 16.0% in 2022, with some of the areas in this province reporting prevalence rates as high as 20.0% [3].

Early initiation of antiretroviral therapy (ART) has reduced morbidity, mortality and HIV transmission [4,5]. In 2015, the World Health Organization (WHO) recommended ART for all PLWH, regardless of the WHO clinical stage or CD4 count, which led to the Universal Test and Treat (UTT) policy being implemented in SA in 2016 [6]. This guidance has contributed to stabilizing HIV incidence, improving survival rates and reducing mortality [1]. Despite these gains a substantial number of individuals in SA continue to present with advanced HIV disease (AHD), defined as having a CD4 cell count below 200 cells/mm^3^ or WHO clinical stage III/IV at ART initiation, indicative of late initiation [7–9]. Following the 2017 WHO recommendations for a comprehensive AHD care package [9], the 2019 SA ART guidelines operationalized a standard package, prioritizing rapid ART initiation and co-infection prophylaxis alongside intensified screening for opportunistic infections. Specifically, the guidelines mandate reflex laboratory screening for cryptococcal antigen (CrAg) for all clients with CD4 < 100 cells/mm^3^, and the use of lateral flow urine lipoarabinomannan (U-LAM) for rapid tuberculosis screening [10]. Baseline CD4 count is key to identifying clients at risk of opportunistic infections, and for understanding the clinical care needs for those who present late [11]. While the national data shows a decline in the prevalence of AHD over time, recent findings indicate that approximately 20.0% of clients present with AHD [2,12]. Moreover, substantial regional disparities persist, with provincial rates ranging between 11.5% and 26.6% in recent years [13,14], underscoring an uneven burden.

Since the introduction of UTT, the number of clients eligible for ART has increased. Adherence to ART reduces onward transmission risk of those living with HIV as well as the probability of CD4 count decline [15]. Therefore, ensuring that PLWH are enrolled and retained in care, and adherent to ART is crucial for better HIV outcomes [16]. Despite this evidence, treatment interruption remains a major challenge, contributing to morbidity, mortality, and transmission [16,17]. Studies conducted in various regions of SA between 2013 and 2022 found that the proportion of clients who experienced disengagement or lost to follow-up from care ranged from 14.7% to 38.8% within two-years of initiating ART [18–20]. These findings highlight that disengagement in care continues to be an important challenge despite expanded access to treatment.

Viral load (VL) monitoring is a key component of ART success, as achieving an undetectable VL is associated with long-term health benefits and eliminates the risk of onward sexual transmission [15]. However, viraemia (often due to poor adherence, or disengagement from care) continues to pose a challenge. South African National Health Laboratory Service (NHLS) data from 2013 to 2022 showed a rise in low-level viraemia VL (50–999 copies/mL), from 15.3% in 2018 to 20.5% in 2022, while high-level viraemia (≥1000 copies/mL) declined during the same period [21]. Viral suppression rates also vary by province; as of 2022, Limpopo had the lowest prevalence of viral suppression (<1000 copies/mL) among adults aged 15 + years (75.3%), and KwaZulu-Natal had the highest (86.7%) [22].

In this study we focused on three persistent challenges that continue to undermine effectiveness of HIV programmes, namely AHD, treatment interruption, and viraemia, aiming to analyse their temporal trends and spatial patterns in three high-burden districts of KwaZulu-Natal, South Africa. The prevalence of these measures varies over time as well as across provinces and sub-districts, making spatial and temporal monitoring critical for informing more focused and effective interventions to improve HIV outcomes.

## Methods

### Ethics statement

This study was approved by the University of KwaZulu-Natal Biomedical Research Ethics Committee (BE646/17), eThekwini Municipality Research Committee, and the KwaZulu-Natal Department of Health’s Provincial Health Research Ethics Committee (KZ_201807_021), with a waiver for informed consent for analysis of anonymized routinely collected data.

### Study design, setting and population

We conducted a retrospective cohort study of clients from 116 primary healthcare clinics in KwaZulu-Natal for which we had data. These clinics are managed by the eThekwini Municipality, uMgungudlovu District, and Mseleni Hospital and Bethesda Hospital in uMkhanyakude District. We included all PLWH aged 16 years and older initiating and collecting ART at participating clinics between 01 July 2018 and 30 June 2023.

HIV care service delivery in South Africa includes the provision of free ART in public healthcare facilities along with a wide range of clinical assessments and laboratory evaluations [10], including VL and CD4 count testing [10,23]. As per South African 2019 guidelines, VL testing was recommended to be done at 6 months on ART, at 12 months if virally suppressed (<50 copies/mL) and annually thereafter for those who remained virally suppressed. For clients with VL ≥ 50 copies/mL, the guidelines recommended that the elevated VLs should be managed with enhanced adherence counselling and repeat of VL testing within 3 months. Meanwhile, CD4 count testing is recommended at initiation and when clinically indicated [10].

### Data source and data management

We analysed routinely collected de-identified electronic health record data from Tier.Net (accessed 19/03/2024), an electronic register used to maintain tuberculosis and HIV client information in public healthcare facilities in South Africa. It contains clients’ demographics, records of ART use, clinic visits, laboratory tests (CD4 counts and VLs), tuberculosis treatment history, and clinical outcomes [24,25].

### Outcomes

Study outcomes were the proportion of clients in a clinic with AHD, treatment interruption, and viraemia, and were measured annually. We estimated AHD proportions using data from all clients initiating ART who had CD4 count data measured within 180 days before to 30 days post ART initiation. Clinical staging data was not available in our dataset, and so only CD4 count <200 cells/mm^3^ was used to define AHD. For clients with multiple CD4 counts within the defined window, we prioritized pre-initiation measurements closest to the initiation date, and then post-initiation measurements.

To evaluate treatment interruption, we included all PLWH on ART who had at least one visit scheduled during the study period. We defined treatment interruption as an unplanned break in HIV care, measured by whether a PLWH missed any scheduled visit by more than 90 days [20,26,27]. We calculated the proportion of clients who experienced at least one treatment interruption in a year. Individuals who were transferred to another clinic or died within 90 days of a scheduled visit were not defined as having an unplanned treatment interruption.

For measurement of viraemia, we included all PLWH who had been on ART for more than 5 months. Among those who had at least one VL result in a year, we calculated the proportion of clients who had VL ≥ 50 copies/mL. If a client had multiple VL tests in a year, the highest VL was selected. Although our main outcome of viraemia was defined using a threshold of 50 copies/mL, in some analyses we additionally report results using VL ≥ 1000 copies/mL in a year.

### Statistical analysis

We used descriptive statistics to summarize clinic-level characteristics in the most recent year using medians and interquartile ranges (IQRs) or counts and percentages. Clinic-level characteristics such as median age and sex distribution were generated by aggregating client-level data annually and per clinic. We estimated clinic size as the number of clients who had at least one visit at that clinic within the study period and assessed socioeconomic context of the clinics using ward-level data. We used the 2011 ward-level South African multidimensional poverty index (SAMPI) from Statistics South Africa to assess area-level deprivation, the most recent census-based metric available at the time of analysis [28]. SAMPI scores range from 0 to 1, where higher scores indicate greater level of deprivation [28]. SAMPI scores were transformed into quintiles based on cut-off values derived from the KwaZulu-Natal ward-level SAMPI distribution, to classify whether clinics are in areas ranging from the least deprived (Q1) to the most deprived (Q5) [29]. Each participating clinic was linked to a ward using its global position system (GPS) coordinates.

We used line plots and bar charts to visualize temporal trends of our study outcomes. To assess the association between clinic-level characteristics and each outcome (AHD, treatment interruption and viraemia (≥50 copies/mL), we used separate log-binomial generalized linear mixed models (GLMM) for each outcome, with results expressed as adjusted relative risks (aRRs) with 95% confidence intervals (CIs). Statistical significance was set at alpha = 0.05. Each outcome model included fixed effects for clinic median age, clinic size quintile, clinic SAMPI quintile, clinic proportion of males and a continuous variable for year of ART initiation/ scheduled visit/ VL testing. Each model also included clinic-specific random effects on the intercept to account for the heterogeneity in outcomes across clinics [30–32] and on year to allow the longitudinal trajectory of the outcomes to vary by clinics. All analyses were performed using R software (version 4.4.0).

### Geospatial mapping

To visualize spatial distribution of each outcome across the five-year period, we calculated crude clinic proportions and generated geospatial maps using QGIS software version 3.40.0 [33]. Mapping was based on a vector layer of clinic records containing GPS coordinates, clinic names, district, ward identifier and proportions of each outcome, and the shapefiles delineating 2018 local municipality boundaries and 2020 ward boundaries in KwaZulu-Natal as base layer of the maps, sourced from the Municipality Demarcation Board [34,35].

We generated inverse distance weighting (IDW) interpolation maps to visualize the proportions of each outcome per district. IDW is a spatial interpolation technique that estimates values at unsampled locations (e.g., areas surrounding the participating clinics) based on known values, assigning weights inversely proportional to the power of the distance between clinics. It operates on the principle that spatial phenomena at proximate locations are more similar than those further apart [36–39].

The algorithm was configured with a default distance coefficient (power parameter) of 2. To preserve the influence of proximate clinics and local variation, we restricted to a maximum of 3 nearest neighbours, with the assumption that clients are most likely to seek care at clinics within their immediate geographic vicinity. This strategy is also supported by literature. Previous research indicates that approximately 90.0% of South African live within 7 km of public clinic, and specifically in eThekwini, 92.0% of the population can access a clinic within 5 km [40,41]. These maps are intended as descriptive and exploratory tools to visualize the raw observed spatial burden at participating clinics, rather than for formal geostatistical inference.

## Results

Among 116 clinics included in analysis, the median clinic size between July 2022 and June 2023 was 4,212 (IQR 2,677, 6,105) clients, Table 1. The median age was 39 (IQR 38, 40) years and the proportion of males was 31.7% (IQR 30.3%, 33.6%). Approximately one in every five clinics (23, 19.8%) were in the most deprived quintiles (Q4 and Q5). Geographically, half of the clinics were located in eThekwini, with 38.8% in uMgungundlovu, and 11.2% in uMkhanyakude. The geographic distribution of clinics across urban, peri-urban and rural is illustrated in the supplementary material (S1 Fig) [36].

**Table 1:** Characteristics of clinics between July 2022 and June 2023.

Characteristic	N = 116, Median [IQR];
**Clinic size**	4,212 [2,677; 6,105]
**Median age**	39.00 [38.00, 40.00]
**Proportion of males**	0.317 [0.303, 0.336]
**Multidimensional Poverty Index quintiles**	**n (%)**
Q1 (least deprived)	46 (39.7%)
Q2	32 (27.6%)
Q3	15 (12.9%)
Q4	13 (11.2%)
Q5 (most deprived)	10 (8.6%)
**District**	**n (%)**
eThekwini	58 (50.0%)
uMgungundlovu	45 (38.8%)
uMkhanyakude	13 (11.2%)

### Advanced HIV disease

**Fig 1:**
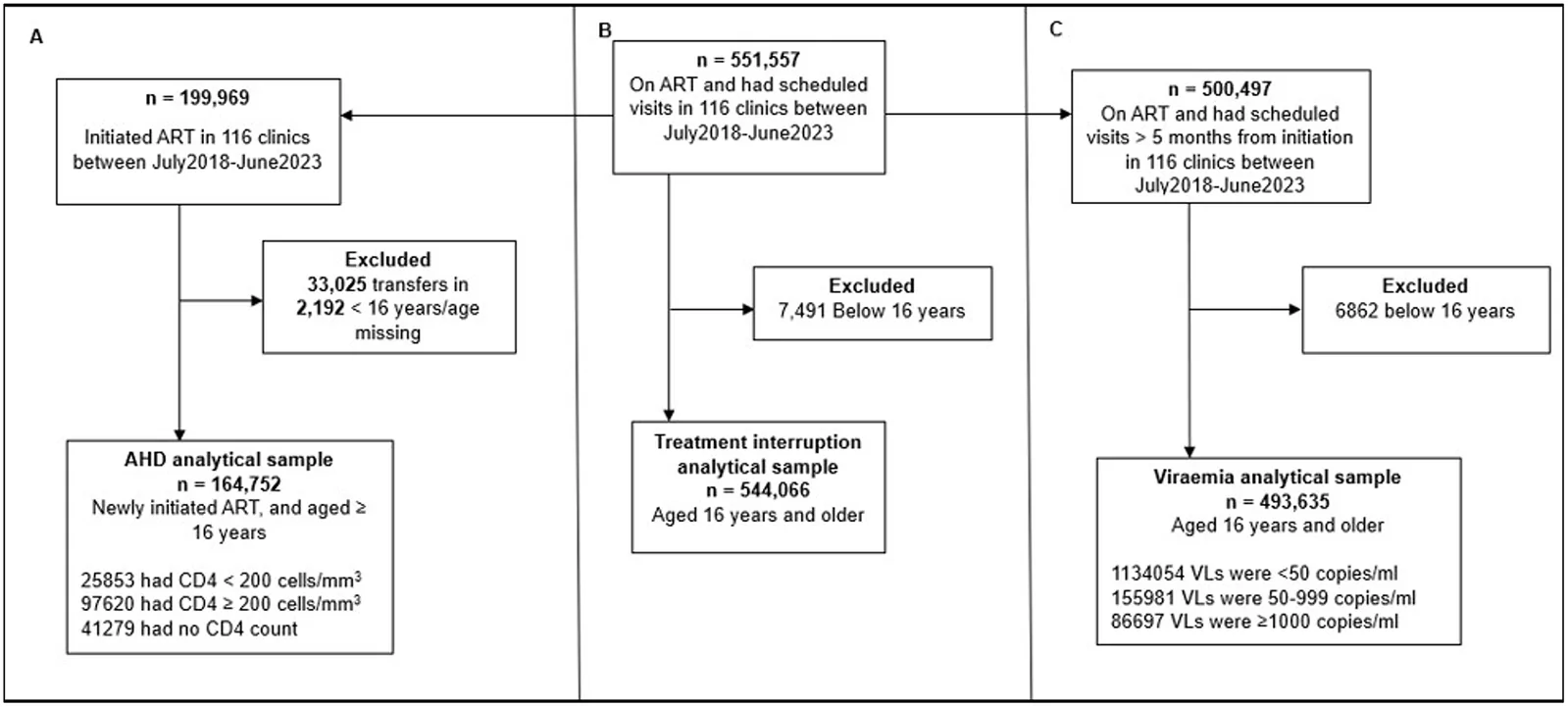
Flowchart for each study outcome. A: advanced HIV disease, B: treatment interruption, and C: viraemia.

**Fig 2:**
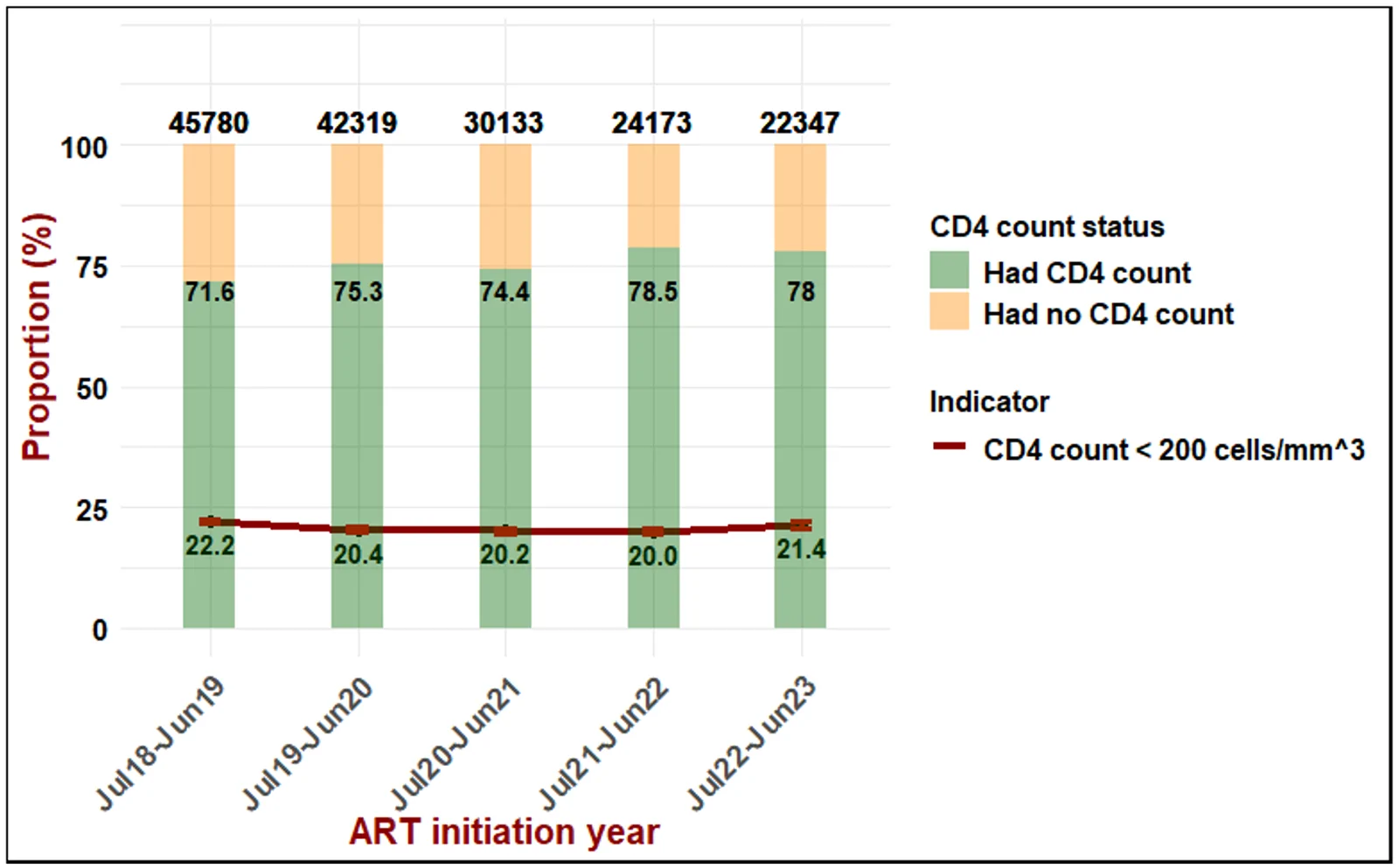
Overall number of ART initiators and prevalence of AHD among clients who had CD4 count across three districts.

The total number of clients who were on ART and included in this study was 551,557 (Fig 1A). We identified 199,969 clients who initiated ART, of whom 164,752 were newly initiated and were included in the AHD analysis. Of these, 41,279 did not have CD4 count data. While the overall number of ART initiations decreased from 45,780 (July2018–June2019) to 22,347 (July2022–June2023), the percentage of clients with CD4 count results recorded at ART initiation improved from 71.6% to 78.0% over the period (Fig 2). The overall prevalence of AHD ranged between 20.0% and 22.2% and between 19.1% and 27.0% at a district level (S2 Fig).

**Fig 3:**
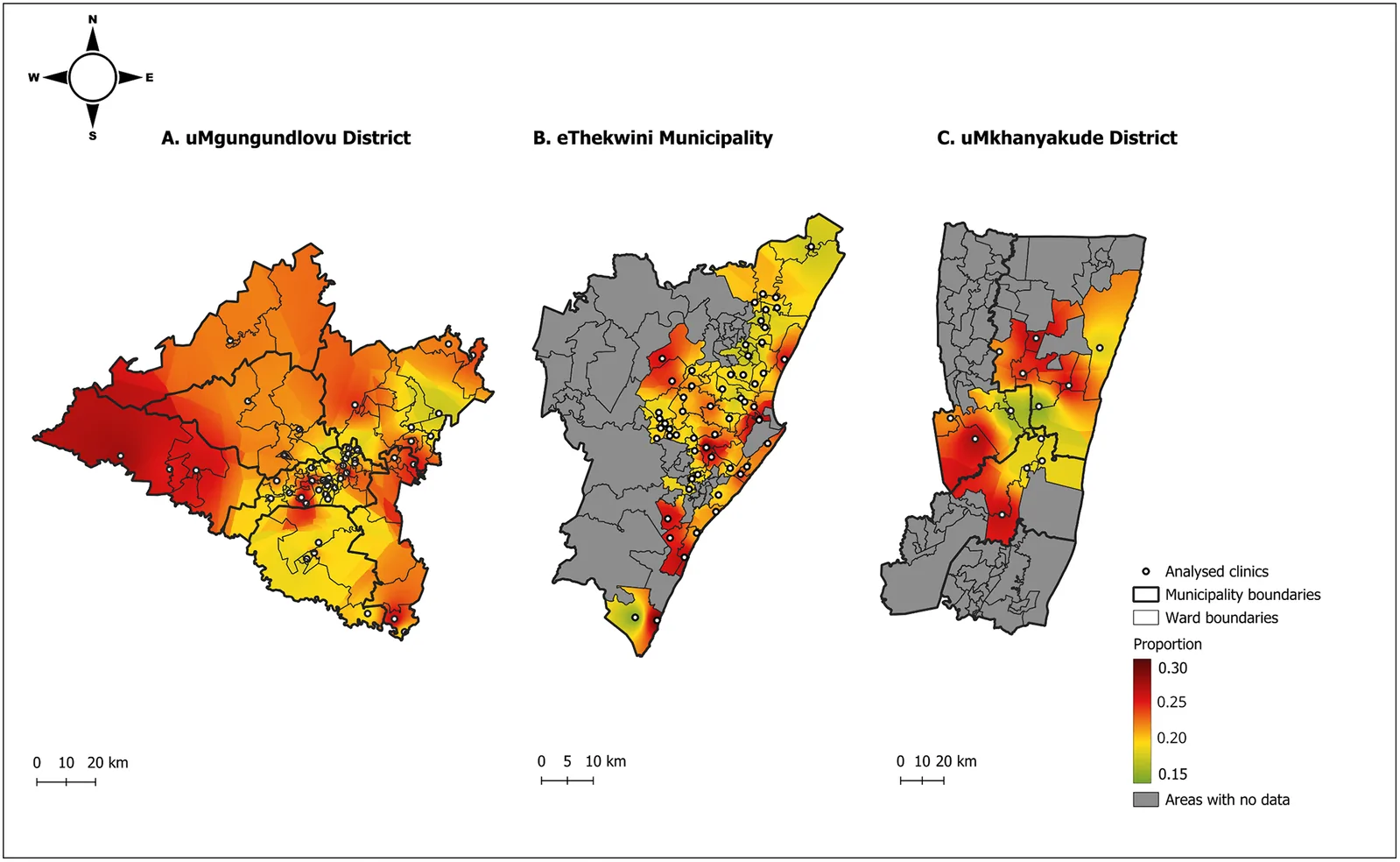
Spatial distribution in prevalence of AHD in three districts of KwaZulu-Natal. The map base layer (wards and local municipality) was obtained from an open-access portal of the South African Municipal Demarcation Board, ward boundaries (MDB Wards 2020 | Municipal Demarcation Board) and local municipality boundaries (MDB Local Municipal Boundary 2018 | Municipal Demarcation Board).

The clinic-level prevalence of AHD across the five-year period ranged from nearly 15.0% to 30.0% (Fig 3) and varied within each district. In uMgungundlovu (Fig 3A), most clinics recorded a prevalence of 20.0% or higher, with notably higher levels in the west (rural) and central (urban) region. In the areas where we had data, elevated prevalence of AHD was observed in the southern and central regions in eThekwini (Fig 3B) and was higher in the northern and southern regions in uMkhanyakude (rural).

On average, the prevalence of AHD in clinics declined slightly over the years (RR:0.98, 95%CI: 0.97-0.99), Table 2. For every one standard deviation (SD) increase (1.84 years) in the median age of clients in a clinic, the risk of AHD increased by 7.0% (RR: 1.07, 95%CI: 1.04-1.09), and for every 1.0% increase in the proportion of males, AHD risk increased by 1.0% (RR: 1.01, 95% CI: 1.00-1.01).

**Table 2:** Adjusted relative risk of clinic-level prevalence of AHD, treatment interruption, viraemia (≥50 copies/mL).

Characteristic	AHD		Treatment interruption		Viraemia (≥50 copies/mL)	
	aRR (95% CI)	p-value	aRR (95% CI)	p-value	aRR (95% CI)	p-value
Median age (standardized)	1.07 (1.04,1.09)	<0.001	0.98 (0.96,1.00)	0.101	0.92 (0.91,0.94)	<0.001
Proportion of male (per 1.0% change)	1.01 (1.00,1.01)	0.001	0.94 (0.93,0.95)	<0.001	1.02 (1.01,1.02)	<0.001
MPI (quintiles)	1.00 (0.98,1.02)	0.981	0.95 (0.91,1.00)	0.031	1.01 (0.98,1.04)	0.610
Clinic size (quintiles)	0.98 (0.97,1.00)	0.074	0.99 (0.95,1.03)	0.585	0.96(0.94,0.99)	0.004
Year	0.98 (0.97,0.99)	0.001	0.99 (0.96,1.01)	0.270	1.12 (1.09,1.14)	<0.001

aRR = adjusted relative risk, CI = confidence interval, MPI = multidimensional poverty index, AHD = advanced HIV disease. Note: The parameter for the median age represents a one standard deviation increase (equivalent to 1.84 years for AHD, 1.71 years for treatment interruption and 1.57 years for viraemia). Proportion of males was grand-mean centered, and the aRR represent the risk associated with a 1.0% increase above the average across all clinics.

### Treatment interruptions

**Fig 4:**
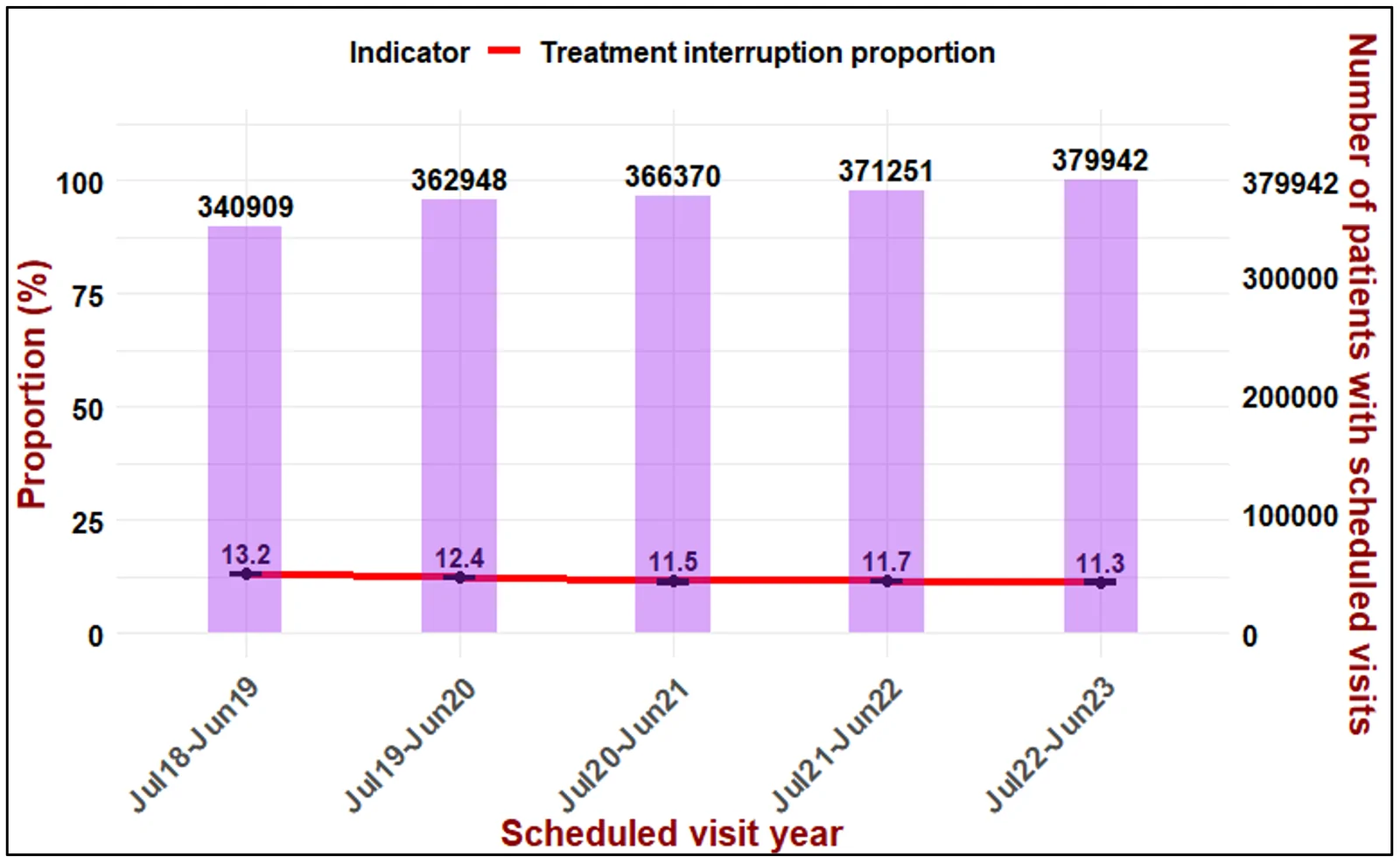
Overall number of PLWH who had scheduled visits and proportion of treatment interruption across three districts.

Among 551,557 clients who were on ART and had at least one scheduled visit during the study period, 544,066 were 16 years or older and were included in the analysis (Fig 1B). The number of clients with scheduled visits increased from 340,909 in July2018–June2019–379,942 in July2022–June2023 (Fig 4). The proportion of treatment interruption was 13.2% in July2018–June2019 and 11.3% by July2022–June2023. At a district level treatment interruption ranged between 10.4% and 13.8% in eThekwini and uMgungundlovu, however in uMkhanyakude it ranged between 11.0% and 15.0% (S4 Fig).

**Fig 5:**
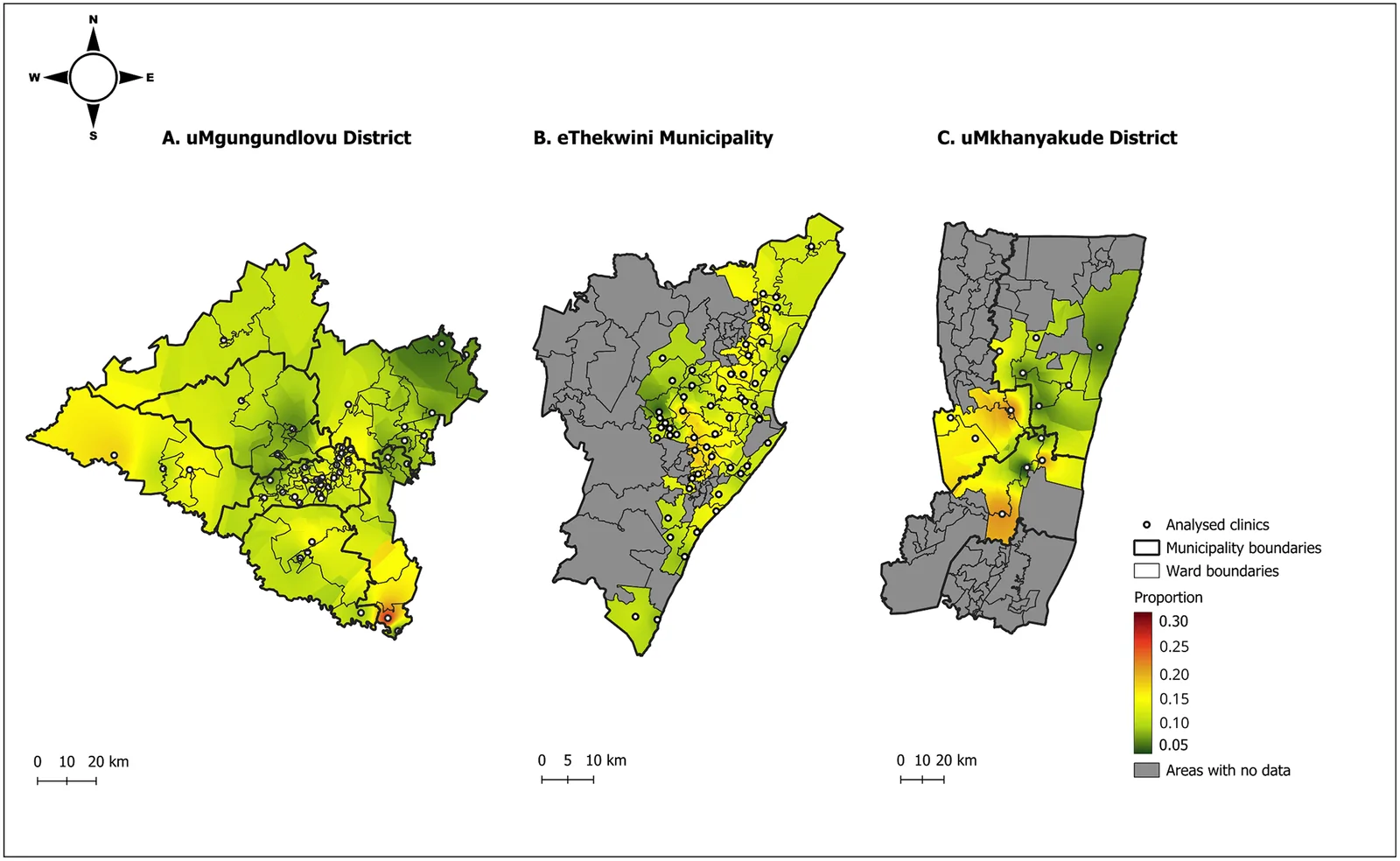
Spatial distribution in the proportion of treatment interruption in three districts of KwaZulu-Natal. The map base layer (wards and local municipality) was obtained from an open-access portal of the South African Municipal Demarcation Board, ward boundaries (MDB Wards 2020 | Municipal Demarcation Board) and local municipality boundaries (MDB Local Municipal Boundary 2018 | Municipal Demarcation Board).

The proportion of treatment interruptions varied across the three districts (Fig 5). In uMgungundlovu, it was generally consistent across clinics, with higher levels of between approximately 15.0% and 30.0% in the western and southern regions (rural). Among the areas we had data for, it was highest in clinics located in the central and parts of the northern region (between 15.0% and 20.0%) in eThekwini. Treatment interruption proportions varied across clinics in the uMkhanyakude. Those located in the western and southern region had the highest levels, ranging from nearly 15.0% to 25.0%.

The GLMM results indicate no evidence of a change in treatment interruption risk in clinics over the study period, however, our findings show that a 1.0% increase in the proportion of males in a clinic was associated with a decrease in the risk of treatment interruption of 6.0% (RR: 0.94, 95%CI: 0.93-0.95), and that increases in the level of economic deprivation was associated with decreased risk of treatment interruption (RR: 0.95, 95%CI: 0.91-1.00), Table 2.

### Viraemia

**Fig 6:**
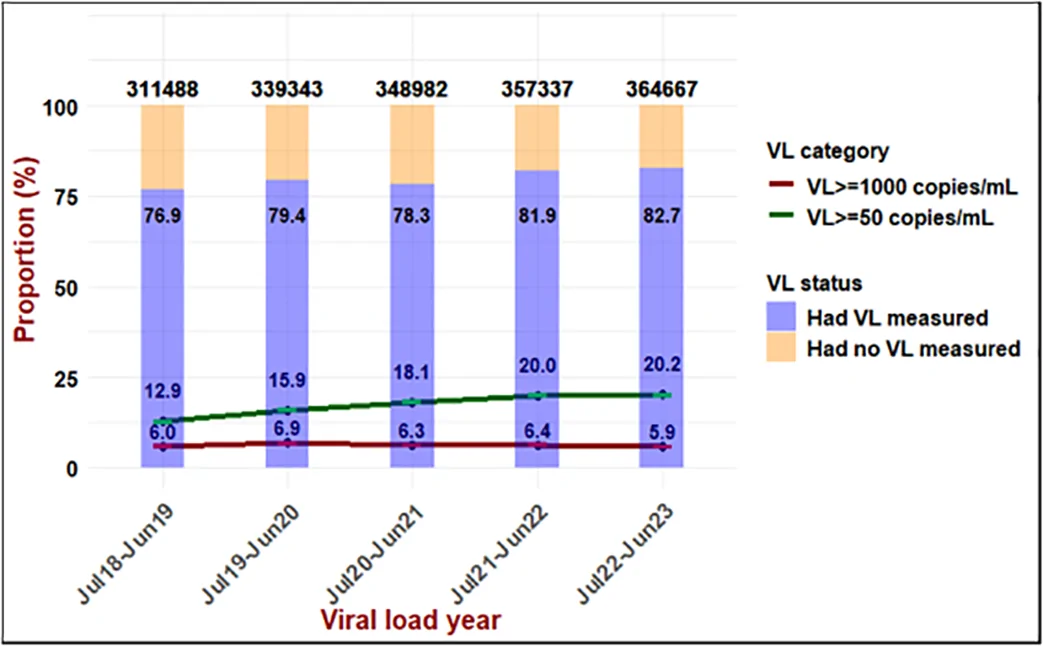
Overall number of PLWH who had scheduled visits more than 5 months from ART initiation and proportion of viraemia ≥50 copies/mL and ≥1000 copies/mL across three districts. VL, viral load; mL, millilitre.

We identified 500,497 clients with at least one scheduled visit more than 5 months from initiation, and 493,635 clients (≥16 years old) were considered for the analysis (Fig 1C). The proportion of clients who had VL results was 76.9% in July2018–June2019 and increased to 82.7% in July2022–June2023 (Fig 6). While the proportion of clients who had high-level viraemia (≥1000 copies/mL) remained stable at approximately 6.0% across all years, the proportion of viraemia (≥50 copies/mL) increased from 12.9% to 20.2%. Overall low-level viraemia (50–999 copies/mL) showed an increase from 6.9% to 14.3% (S6 Fig). The increase in viraemia (≥50 copies/mL) and low-level viraemia (50–999 copies/mL) was most pronounced in eThekwini district (S6–S7 Figs).

**Fig 7:**
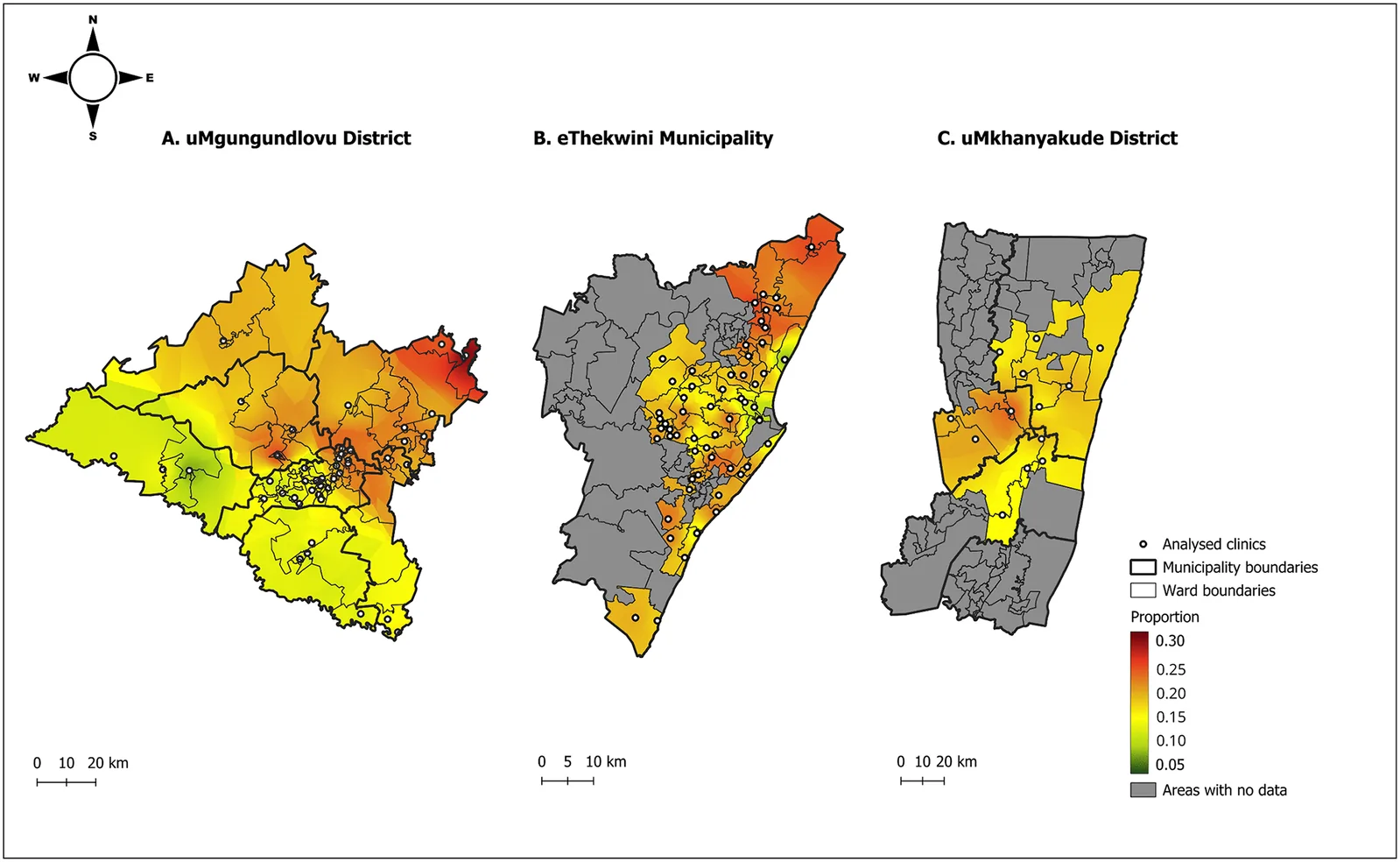
Spatial distribution in the proportion of viraemia (≥50 copies/mL) in three districts of KwaZulu-Natal. The map base layer (wards and local municipality) was obtained from an open-access portal of the South African Municipal Demarcation Board, ward boundaries (MDB Wards 2020 | Municipal Demarcation Board) and local municipality boundaries (MDB Local Municipal Boundary 2018 | Municipal Demarcation Board).

High proportions of viraemia (≥50 copies/mL) were observed in clinics located in the central (urban) and north-eastern (peri-urban) region of uMgungundlovu ranging from around 15.0% to 30.0%, and in the northern, central and southern region of eThekwini between 15.0% and 25.0% (Fig 7) in areas where we had data. The prevalence of viraemia was similar across clinics examined in uMkhanyakude.

On average, the proportion of viraemia (≥50 copies/mL) in clinics, increased significantly over the years (RR: 1.12, 95%CI: 1.09-1.14), Table 2. Clinics serving older clients (1.57 years increase in median age) had a reduced risk of viraemia (RR: 0.92, 95% CI: 0.91-0.94), while an increase in the proportion of males (1.0% increase from the average male proportion) was associated with an increased risk (RR:1.02 95%CI:1.01-1.02). However, an increase in the size of clinics was associated with a reduced risk (RR: 0.96, 95% CI: 0.94-0.99).

## Discussion

In this study, we examined temporal and spatial variation in the prevalence of AHD, treatment interruption, and viraemia, three key measures of HIV programmatic performance, using data from the KwaZulu-Natal province in South Africa between 2018 and 2023. We also explored whether temporal changes in prevalence are associated with clinic characteristics such as median clinic age, sex proportions, clinic size and clinic area wealth quintiles. The findings of this analysis can be used to inform more targeted interventions to improve HIV service delivery in KwaZulu-Natal and other hyper-endemic settings.

We found that AHD prevalence remained high over the five-year period at approximately 20.0–22.0%, aligning with recent studies reporting AHD between 19.5% to 20.8% at a national and between 11.6% and 28.6% provincial level between 2017 and 2023 [12,42]. However, our analysis of temporal trajectory revealed that on average, the prevalence of AHD has declined slightly over the years in clinics. In a clinic-level analysis, we found that an increase in proportion of males and aging of clients in clinics was associated with increased risk of AHD. This is consistent with other studies where older age and male sex were reported to be at high risk [2,42]. Gender disparity is likely due to men’s lower engagement in HIV testing and treatment, driven by stigma and suboptimal health-seeking behaviors [43,44]. Taken together, these results highlight that despite improved ART access, late presentation remains a challenge. To further reduce AHD among ART naïve clients, strategies must be more targeted and focus on early HIV testing and linkage to care, especially among men [42].

Our definition of treatment interruption aligns with commonly used measures of disengagement from care [20,26,27,45,46], however some studies have used longer thresholds than ours (e.g., > 180 days late for a visit) [18,47]. Studies have also assessed loss-to-follow-up at specified follow-up intervals since ART initiation (e.g., at 6, 12, 18, 24 months or more) [17,26,27,46], whereas our analysis examined interruptions over time, and regardless of prior interruptions and time on ART. In the literature, prevalence of disengagement from care varied between studies done in SA, ranging between 14.7% and 50.3% depending on the time on ART [18,20,27,46], and period used for analysis. In our study, prevalence of treatment interruption remained persistent ranging between 11.3% and 13.2%. Though this is lower than previous studies, we noted that the prevalence of treatment interruptions differed across and within districts, with some areas showing prevalence of up to approximately 30.0%. We found no evidence of a decline in prevalence during the observation period. Interestingly, we observed that an increase in the proportion of male clients in a clinic was associated with reduced risk of interruption. These findings differ from the existing literature reported in SA and elsewhere, where male sex tends to increase the risk of disengagement from care [16,18,20]. Additionally, we found that higher levels of poverty were associated with reduced risk of interruption, an association that contrasts with the traditional view of socioeconomic deprivation as barrier to retention. These unexpected associations could reflect inaccuracies introduced by using the outdated deprivation index, or be caused by aggregating data to the clinic-level, as clinic-level patterns may not align with individual-level ones [48]. A study conducted in SA between 2014 and 2018 showed that males initiating ART at male-focused clinics had reduced risk of dropping out of care [44], giving support to the idea that retention of males is better in clinics with a greater proportion of males, and can improve overall clinic performance.

Over the five-year period, we observed an increase in the proportion of clients with documented VL results from 76.9% to 82.7%. This progress indicates an improvement in clinical VL monitoring despite increasing number of clients on ART. However, the persistent gap in missing VL tests needs further investigation as part of managing viraemia within the province. In this cohort, we observed an overall increase in viraemia (≥50 copies/mL), rising from 12.9% to 20.2% and low-level viraemia (50–999 copies/mL) from 6.9% to 14.3%. Our GLMM findings confirmed that over the period the increase in viraemia (≥50 copies/mL) in clinics was significant. In contrast, viraemia (≥1000 copies/mL) remained stable at approximately 6.0%. A similar trend of increasing rates of low-level VL (50–999 copies/mL) both at regional and national level, alongside a decline in high-level viraemia over time has been reported by recent studies [21,36]. These findings may reflect promising improvements in preventing sustained high-level viraemia, partly attributable to the roll-out of dolutegravir-based treatment in late 2019 [10,49], while highlighting a growing burden of low-level viraemia. Finally, we noted that large clinics serving older clients were associated with reduced viraemia (≥50 copies/mL) risk, while those with a higher proportion of males had greater prevalence of viraemia (≥50 copies/mL). Our findings are in line with previous studies reporting that younger age is associated with an increased risk of viral rebound [50,51], and that male sex was associated with increased risk of viraemia [51,52].

The results from our spatial analysis revealed that the burden of AHD, treatment interruptions, and viraemia (≥50 copies/mL) varies across and within the studied districts, spanning urban, peri-urban and rural areas. In rural communities such as parts of uMgungundlovu and uMkhanyakude districts, these challenges may be attributed to disparate access to HIV testing [53] delaying early access to ART, and declines in ART initiations during COVID-19 pandemic. Structural barriers such as geographic isolation, supply stockouts and long travel distances to clinics may also contribute [54]. Whereas, in high-density urban settings within eThekwini and uMgungundlovu, these challenges may be driven by higher prevalence of HIV [54], clinic congestion, informal settlement dynamics, and high levels of migration for employment which disrupt care continuity.

The size of our cohort (incorporating approximately half a million people on ART, drawn from both rural and urban public sector settings) and the length of the longitudinal data employed enhances the generalizability of our findings and is a key strength of the analysis. However, there are limitations to the use of TIER.Net data that must be highlighted. “Silent transfers” are not captured in TIER.Net and will have led to an over-estimation of treatment interruptions and misclassification of ART naïve clients [55]. VL and CD4 count data in TIER.Net is also not complete, although a recent study showed that the VL data quality has improved across all levels of VL [56]. Although CD4 count data remains under-captured, the degree of under-capture has not been associated with CD4 count level and therefore should not impact on the interpretation of our findings [56].

Geospatial mapping was used as a descriptive tool to visualize observed spatial burdens across participating clinics. However, these findings should be interpreted with caution due to several challenges. First, the analysis relied on clinic coordinates rather than client residential area. Clients residential location could have supported a more accurate estimation of clinic catchment areas and allowed us to assess spatial autocorrelation beyond a distance (such as directional trends or physical geographic barriers). Nonetheless, the use of clinic locations remains valuable, by identifying where healthcare system experiences the highest burden. Secondly, the use of the IDW method introduces specific limitations. In particular, the underlying algorithm relies on the distance between each sampled clinic and the unsampled point, it does not adjust for covariates, lacks the capacity to generate prediction intervals to quantify estimation uncertainty, and fails to account for directional trends. The method is also prone to the “bull’s eye” effect around isolated sampled points, where the measured point only influences a small area around it, which does not reflect actual geographic transition [57]. All maps (Figs 3, 5 and 7) are overlayed with locations of the clinics used in the analysis to assist readers in identifying isolated clinics where uncertainty and interpolation artifacts are more likely to occur.

## Conclusion

In this study we demonstrated that AHD, treatment interruptions viraemia continue to pose a challenge to reducing HIV burden in SA. Crucially, our findings also revealed geographic heterogeneity in these measures. The persistent burden of AHD, increasing viraemia, and marked spatial variation across the studied districts underscores the need for continued geospatial analysis. By uncovering these localized disparities, this study highlights the unique utility of geospatial analysis as a tool for near real-time epidemiology surveillance, providing the empirical foundation to design geographically tailored interventions to strengthen programmatic effectiveness in hyper-endemic settings like KwaZulu-Natal.

## Supporting information

S1 Fig Study area, sub-districts and clinic locations. The map base layer was obtained from an open-access portal of the South African Municipal Demarcation Board (MDB Local Municipal Boundary 2018 | Municipal Demarcation Board).
(TIF)

S2 Fig Overall number of ART initiators and prevalence of AHD among clients who had CD4 count by district. (TIF)

S3 Fig Overall number of ART initiators and prevalence of AHD among clients who had CD4 count by sub-district. (TIF)

S4 Fig Overall number of PLWH who had scheduled visits and proportion of treatment interruption by district. (TIF)

S5 Fig Overall number of PLWH who had scheduled visits and proportion of treatment interruption by sub-district. (TIF)

S6 Fig Overall number of PLWH who had scheduled visits more than 5 months from ART initiation and proportion of low-level viraemia. VL, viral load; mL, millilitre.
(TIF)

S7 Fig Overall number of PLWH who had scheduled visits more than 5 months from ART initiation and proportion of low-level viraemia by district. VL, viral load; mL, millilitre.
(TIF)

S8 Fig Overall number of PLWH who had scheduled visits more than 5 months from ART initiation and proportion of viraemia by district. VL, viral load; mL, millilitre.
(TIF)

S9 Fig Overall number of PLWH who had scheduled visits more than 5 months from ART initiation and proportion of viraemia by sub-district. VL, viral load; mL, millilitre.
(TIF)

S1 Table Characteristics of clients by year and CD4 count category at ART initiation. (DOCX)

S2 Table Characteristics of clients by scheduled visit year and treatment interruption status at first visit in each year. (DOCX)

S3 Table Characteristics of clients by viral load year and viral load category at viral load testing. (DOCX)

S4 Table Unadjusted relative risk of clinic-level prevalence of AHD, treatment interruption, viraemia. (DOCX)

S5 Table Estimates of random effects for AHD, treatment interruption, and viraemia. (DOCX)

## Abbreviations

AHD: Advanced HIV disease
ART: Antiretroviral therapy
HIV: Human immunodeficiency virus
PLWH: People living with HIV
TIER.Net: Three Interlinked Electronic Register
UTT: Universal test and treat
VL: Viral load.
