# Supplementary material for: Trends in advanced HIV disease, treatment interruption, and viraemia in KwaZulu-Natal, South Africa
Source: PLOS Glob Public Health. 2026 Sep 2;6(9):e0005506. doi: 10.1371/journal.pgph.0005506 (PMC13537683; doi:10.1371/journal.pgph.0005506)
Supplement: Supplementary file 1 [file pgph.0005506.s010.docx]

| **Characteristic** | **Jul18-Jun19** | | **Jul19-Jun20** | | **Jul20-Jun21** | | **Jul21-Jun22** | | **Jul22-Jun23** | |
| --- | --- | --- | --- | --- | --- | --- | --- | --- | --- | --- |
|  | **CD4<200** N = 7,292*^1^* | **CD4 >=200** N = 25,507*^1^* | **CD4 <200** N = 6,503*^1^* | **CD4 >=200** N = 25,353*^1^* | **CD4 <200** N = 4,534*^1^* | **CD4 >=200** N = 17,880*^1^* | **CD4 <200** N = 3,792*^1^* | **CD4 >=200** N = 15,182*^1^* | **CD4 <200** N = 3,731*^1^* | **CD4 >=200** N = 13,698*^1^* |
| **Age (years)** | 34 (29, 41) | 31 (25, 37) | 35 (29, 41) | 31 (25, 37) | 34 (29, 41) | 30 (25, 37) | 35 (30, 41) | 31 (25, 37) | 36 (30, 42) | 31 (26, 38) |
| **Age categories** |  |  |  |  |  |  |  |  |  |  |
| 16-25 | 856 (11.5%) | 6,571 (88.5%) | 742 (10.1%) | 6,621 (89.9%) | 543 (10.3%) | 4,712 (89.7%) | 438 (10.2%) | 3,876 (89.8%) | 399 (10.8%) | 3,295 (89.2%) |
| 26-35 | 3,265 (22.2%) | 11,436 (77.8%) | 2,824 (20.2%) | 11,187 (79.8%) | 1,961 (19.7%) | 8,013 (80.3%) | 1,493 (18.3%) | 6,666 (81.7%) | 1,430 (19.7%) | 5,844 (80.3%) |
| 36-45 | 2,200 (29.6%) | 5,240 (70.4%) | 2,029 (28.2%) | 5,172 (71.8%) | 1,409 (28.1%) | 3,603 (71.9%) | 1,268 (28.2%) | 3,235 (71.8%) | 1,250 (28.7%) | 3,106 (71.3%) |
| 46+ | 971 (30.1%) | 2,260 (69.9%) | 908 (27.7%) | 2,373 (72.3%) | 621 (28.6%) | 1,552 (71.4%) | 593 (29.7%) | 1,405 (70.3%) | 652 (31.0%) | 1,453 (69.0%) |
| **Sex** |  |  |  |  |  |  |  |  |  |  |
| Female | 3,632 (17.0%) | 17,680 (83.0%) | 3,210 (16.0%) | 16,874 (84.0%) | 2,247 (15.6%) | 12,161 (84.4%) | 1,841 (15.2%) | 10,282 (84.8%) | 1,758 (16.1%) | 9,139 (83.9%) |
| Male | 3,660 (31.9%) | 7,827 (68.1%) | 3,293 (28.0%) | 8,479 (72.0%) | 2,287 (28.6%) | 5,719 (71.4%) | 1,951 (28.5%) | 4,900 (71.5%) | 1,973 (30.2%) | 4,559 (69.8%) |
| **CD4 Count (Cells/mm^3^)** | 110 (60, 150) | 450 (320, 630) | 110 (60, 160) | 460 (320, 640) | 110 (60, 160) | 460 (330, 650) | 110 (60, 160) | 470 (330, 670) | 110 (60, 160) | 460 (330, 650) |
| **District** |  |  |  |  |  |  |  |  |  |  |
| eThekwini Metropolitan Municipality | 4,861 (22.1%) | 17,112 (77.9%) | 4,295 (20.5%) | 16,608 (79.5%) | 2,924 (20.3%) | 11,481 (79.7%) | 2,353 (19.1%) | 9,987 (80.9%) | 2,366 (20.6%) | 9,145 (79.4%) |
| uMgungundlovu District Municipality | 2,099 (22.7%) | 7,134 (77.3%) | 1,858 (19.7%) | 7,576 (80.3%) | 1,355 (19.9%) | 5,455 (80.1%) | 1,207 (21.8%) | 4,340 (78.2%) | 1,116 (22.3%) | 3,879 (77.7%) |
| uMkhanyakude District Municipality | 332 (20.8%) | 1,261 (79.2%) | 350 (23.0%) | 1,169 (77.0%) | 255 (21.3%) | 944 (78.7%) | 232 (21.3%) | 855 (78.7%) | 249 (27.0%) | 674 (73.0%) |
| *^1^* Median (Q1, Q3); n (%) | | | | | | | | | | |
