# Supplementary material for: Trends in advanced HIV disease, treatment interruption, and viraemia in KwaZulu-Natal, South Africa
Source: PLOS Glob Public Health. 2026 Sep 2;6(9):e0005506. doi: 10.1371/journal.pgph.0005506 (PMC13537683; doi:10.1371/journal.pgph.0005506)
Supplement: Supplementary file 2 [file pgph.0005506.s011.docx]

| **Characteristic** | **Jul18-Jun19** | | **Jul19-Jun20** | | **Jul20-Jun21** | | **Jul21-Jun22** | | **Jul22-Jun23** | |
| --- | --- | --- | --- | --- | --- | --- | --- | --- | --- | --- |
|  | **No interruption**  N = 296,063*^1^* | **Had interruption** N = 44,846*^1^* | **No interruption** N = 317,951*^1^* | **Had interruption** N = 44,996*^1^* | **No interruption** N = 324,352*^1^* | **Had interruption** N = 42,017*^1^* | **No interruption** N = 327,918*^1^* | **Had interruption** N = 43,332*^1^* | **No interruption** N = 336,900*^1^* | **Had interruption** N = 43,041*^1^* |
| **Age (years)** | 36 (30, 44) | 32 (27, 39) | 37 (31, 45) | 32 (27, 39) | 38 (31, 45) | 33 (27, 40) | 38 (32, 46) | 34 (28, 41) | 39 (33, 47) | 35 (29, 42) |
| **Age categories** |  |  |  |  |  |  |  |  |  |  |
| 16-25 | 31,471 (78.2%) | 8,781 (21.8%) | 31,038 (77.0%) | 9,278 (23.0%) | 28,654 (79.6%) | 7,365 (20.4%) | 26,538 (79.7%) | 6,765 (20.3%) | 23,854 (79.5%) | 6,166 (20.5%) |
| 26-35 | 105,897 (84.5%) | 19,363 (15.5%) | 107,854 (84.8%) | 19,317 (15.2%) | 104,664 (85.9%) | 17,220 (14.1%) | 99,824 (85.5%) | 16,990 (14.5%) | 94,092 (85.3%) | 16,270 (14.7%) |
| 36-45 | 95,330 (89.4%) | 11,276 (10.6%) | 105,752 (90.5%) | 11,051 (9.5%) | 110,136 (90.6%) | 11,441 (9.4%) | 113,625 (90.0%) | 12,607 (10.0%) | 119,292 (89.8%) | 13,479 (10.2%) |
| 46+ | 63,365 (92.1%) | 5,426 (7.9%) | 73,307 (93.2%) | 5,350 (6.8%) | 80,898 (93.1%) | 5,991 (6.9%) | 87,931 (92.7%) | 6,970 (7.3%) | 99,662 (93.3%) | 7,126 (6.7%) |
| **Sex** |  |  |  |  |  |  |  |  |  |  |
| Female | 209,054 (88.7%) | 26,681 (11.3%) | 218,699 (88.3%) | 29,050 (11.7%) | 221,752 (89.1%) | 27,124 (10.9%) | 224,628 (89.2%) | 27,222 (10.8%) | 231,062 (89.8%) | 26,297 (10.2%) |
| Male | 87,009 (82.7%) | 18,165 (17.3%) | 99,252 (86.2%) | 15,946 (13.8%) | 102,600 (87.3%) | 14,893 (12.7%) | 103,290 (86.5%) | 16,110 (13.5%) | 105,838 (86.3%) | 16,744 (13.7%) |
| **District** |  |  |  |  |  |  |  |  |  |  |
| eThekwini Metropolitan Municipality | 173,373 (86.2%) | 27,740 (13.8%) | 186,722 (86.8%) | 28,296 (13.2%) | 191,983 (88.1%) | 25,849 (11.9%) | 194,814 (88.1%) | 26,283 (11.9%) | 204,596 (89.2%) | 24,697 (10.8%) |
| uMgungundlovu District Municipality | 102,390 (87.4%) | 14,748 (12.6%) | 110,574 (89.1%) | 13,469 (10.9%) | 111,636 (89.8%) | 12,722 (10.2%) | 111,681 (89.0%) | 13,734 (11.0%) | 110,427 (88.0%) | 15,056 (12.0%) |
| uMkhanyakude District Municipality | 20,300 (89.6%) | 2,358 (10.4%) | 20,655 (86.5%) | 3,231 (13.5%) | 20,733 (85.7%) | 3,446 (14.3%) | 21,423 (86.6%) | 3,315 (13.4%) | 21,877 (86.9%) | 3,288 (13.1%) |
| *^1^* Median (Q1, Q3); n (%) | | | | | | | | | | |
