# Supplementary material for: Trends in advanced HIV disease, treatment interruption, and viraemia in KwaZulu-Natal, South Africa
Source: PLOS Glob Public Health. 2026 Sep 2;6(9):e0005506. doi: 10.1371/journal.pgph.0005506 (PMC13537683; doi:10.1371/journal.pgph.0005506)
Supplement: Supplementary file 3 [file pgph.0005506.s012.docx]

| **Characteristic** | **Jul18-Jun19** | | **Jul19-Jun20** | | **Jul20-Jun21** | | **Jul21-Jun22** | | **Jul22-Jun23** | |
| --- | --- | --- | --- | --- | --- | --- | --- | --- | --- | --- |
|  | **VL <50 copies/mL** N = 208,808*^1^* | **VL >=50 copies/mL** N = 30,796*^1^* | **VL<50 copies /mL** N = 226,543*^1^* | **VL >=50 copies/mL** N = 42,870*^1^* | **VL <50 copies/mL** N = 223,830*^1^* | **VL >=50 copies/mL** N = 49,556*^1^* | **VL <50 copies/mL** N = 233,947*^1^* | **VL >=50 copies/mL** N = 58,633*^1^* | **VL <50 copies/mL** N = 240,926*^1^* | **VL >=50 copies/mL** N = 60,821*^1^* |
| **Age (years)** | 38 (31, 45) | 36 (29, 43) | 38 (32, 46) | 36 (30, 44) | 39 (32, 46) | 37 (31, 45) | 39 (33, 47) | 39 (32, 46) | 40 (33, 48) | 39 (33, 47) |
| **Age categories** |  |  |  |  |  |  |  |  |  |  |
| 16-25 | 16,215 (79.8%) | 4,101 (20.2%) | 16,781 (75.3%) | 5,492 (24.7%) | 15,856 (74.3%) | 5,489 (25.7%) | 14,978 (73.0%) | 5,535 (27.0%) | 13,799 (72.5%) | 5,242 (27.5%) |
| 26-35 | 70,635 (86.5%) | 11,056 (13.5%) | 72,776 (83.0%) | 14,858 (17.0%) | 68,667 (81.2%) | 15,875 (18.8%) | 67,120 (80.1%) | 16,701 (19.9%) | 64,367 (80.3%) | 15,806 (19.7%) |
| 36-45 | 71,949 (87.8%) | 9,967 (12.2%) | 79,195 (85.1%) | 13,843 (14.9%) | 78,321 (82.4%) | 16,775 (17.6%) | 83,467 (80.4%) | 20,332 (19.6%) | 87,172 (80.0%) | 21,859 (20.0%) |
| 46+ | 50,009 (89.8%) | 5,672 (10.2%) | 57,791 (86.9%) | 8,677 (13.1%) | 60,986 (84.2%) | 11,417 (15.8%) | 68,382 (81.0%) | 16,065 (19.0%) | 75,588 (80.8%) | 17,914 (19.2%) |
| **Sex** |  |  |  |  |  |  |  |  |  |  |
| Female | 146,119 (88.3%) | 19,293 (11.7%) | 158,233 (85.5%) | 26,878 (14.5%) | 156,858 (83.3%) | 31,342 (16.7%) | 164,297 (81.5%) | 37,232 (18.5%) | 168,938 (81.5%) | 38,366 (18.5%) |
| Male | 62,689 (84.5%) | 11,503 (15.5%) | 68,310 (81.0%) | 15,992 (19.0%) | 66,972 (78.6%) | 18,214 (21.4%) | 69,650 (76.5%) | 21,401 (23.5%) | 71,988 (76.2%) | 22,455 (23.8%) |
| **Viral load (cp/ml)** | 0 (0, 10) | 710 (120, 12,800) | 10 (0, 10) | 550 (110, 9,320) | 10 (10, 10) | 340 (100, 3,580) | 10 (10, 10) | 340 (110, 2,020) | 10 (10, 10) | 270 (100, 1,560) |
| **District** |  |  |  |  |  |  |  |  |  |  |
| eThekwini Metropolitan Municipality | 129,657 (88.8%) | 16,329 (11.2%) | 139,475 (85.6%) | 23,495 (14.4%) | 134,727 (81.4%) | 30,789 (18.6%) | 139,494 (79.4%) | 36,250 (20.6%) | 142,191 (76.8%) | 43,074 (23.2%) |
| uMgungundlovu District Municipality | 64,506 (83.9%) | 12,347 (16.1%) | 71,700 (81.8%) | 15,981 (18.2%) | 73,414 (83.2%) | 14,855 (16.8%) | 77,635 (80.4%) | 18,955 (19.6%) | 81,545 (85.4%) | 13,904 (14.6%) |
| uMkhanyakude District Municipality | 14,645 (87.4%) | 2,120 (12.6%) | 15,368 (81.9%) | 3,394 (18.1%) | 15,689 (80.0%) | 3,912 (20.0%) | 16,818 (83.1%) | 3,428 (16.9%) | 17,190 (81.7%) | 3,843 (18.3%) |
| *^1^* Median (Q1, Q3); n (%), VL = viral load, mL = Millilitre | | | | | | | | | | |
