# Supplementary material for: Trends in advanced HIV disease, treatment interruption, and viraemia in KwaZulu-Natal, South Africa
Source: PLOS Glob Public Health. 2026 Sep 2;6(9):e0005506. doi: 10.1371/journal.pgph.0005506 (PMC13537683; doi:10.1371/journal.pgph.0005506)
Supplement: Supplementary file 4 [file pgph.0005506.s013.docx]

| **Characteristic** | **AHD** | | **Treatment interruption** | | **Viraemia** | |
| --- | --- | --- | --- | --- | --- | --- |
|  | **RR** **(95% CI**) | **P-value** | **RR** (**95% CI**) | **P-value** | **RR** **(95% CI**) | **P-value** |
| Median age(standardized) | 1.08 (1.06, 1.10) | <0.001 | 0.98 (0.95,1.00) | 0.038 | 0.92 (0.91,0.94) | <0.001 |
| Proportion of male (per 1% change) | 1.01 (1.01, 1.01) | <0.001 | 0.94 (0.93, 0.95) | <0.001 | 1.02 (1.01,1.02) | <0.001 |
| Multidimensional Poverty Index(quintiles) | 0.98 (0.97, 1.00) | 0.060 | 0.98 (0.94,1.02) | 0.227 | 1.02 (0.99,1.05) | 0.219 |
| Clinic size(quintiles) | 0.98 (0.96, 1.00) | 0.039 | 1.00 (0.96,1.04) | 0.938 | 0.97 (0.94,0.99) | 0.015 |

RR = Relative Risk, CI = Confidence Interval, AHD = advanced HIV disease

**Model Estimation**

For all three models, we estimated parameters using Maximum Likelihood with Laplace Approximation using the glmer() function within the lme4 package. To ensure numerical stability and valid convergence of the log-binomial generalized linear mixed models (GLMMs), models were fit using the Bound Optimization by Quadratic Approximation (BOBYQA) optimizer with default settings. Furthermore, to reduce eigenvalue disparities and facilitate stable optimization, we applied data transformation techniques, the median age was standardized using Z-scores, the proportion of male clients was mean-centered, and both clinic size and the multidimensional poverty index were modeled as quintiles. Random effects are summarized by standard deviations (SDs) of clinic-specific intercepts and slopes, alongside their intercept-slope correlations. To quantify uncertainty of the random effects, 95% Profile likelihood CIs for random effects were obtained confint() function in the lme4 package.
