# Supplementary material for: Trends in advanced HIV disease, treatment interruption, and viraemia in KwaZulu-Natal, South Africa
Source: PLOS Glob Public Health. 2026 Sep 2;6(9):e0005506. doi: 10.1371/journal.pgph.0005506 (PMC13537683; doi:10.1371/journal.pgph.0005506)
Supplement: Supplementary file 5 [file pgph.0005506.s014.docx]

| **Random Effect Term** | **SD (95% CI)** |
| --- | --- |
| **AHD** |  |
| Random intercept (clinic) | 0.106 (0.069, 0.145) |
| Random slope (year by clinic) | 0.037 (0.026, 0.050) |
| Intercept-year correlation | -0.580 (-0.778, -0.172) |
|  | |
| **Treatment interruption** |  |
| Random intercept (clinic) | 0.426 (0.374, 0.490) |
| Random slope (schedule visit year by clinic) | 0.120 (0.105, 0.137) |
| Intercept-year correlation | -0.693 (-0.779, -0.582) |
|  | |
| **Viraemia (>=50 copies/mL)** |  |
| Random intercept (clinic) | 0.345 (0.303, 0.398) |
| Random slope (VL year by clinic) | 0.119 (0.104, 0.137) |
| Intercept-year correlation | -0.840 (-0.888, -0.774) |
