# Supplementary material for: Trends in advanced HIV disease, treatment interruption, and viraemia in KwaZulu-Natal, South Africa
Source: PLOS Glob Public Health. 2026 Sep 2;6(9):e0005506. doi: 10.1371/journal.pgph.0005506 (PMC13537683; doi:10.1371/journal.pgph.0005506)
Supplement: Supplementary file 6 [file pgph.0005506.s015.docx]

Trends in advanced HIV disease, treatment interruption, and viraemia in KwaZulu- Natal, South Africa: PGPH-D-25-03407

This manuscript makes a substantive contribution to HIV program monitoring by integrating longitudinal routine data with spatial visualization to analyze advanced HIV disease (AHD), treatment interruption, and viraemia. The study leverages a rich, programmatically relevant dataset ([Tier.net](https://tier.net/)) and addresses critical questions for hyper-endemic settings like KwaZulu-Natal. While the core regression methodology is strong, the spatial analysis component could be enhanced to move beyond descriptive visualization toward model-based inference.

Verdict: **Major Revision**

The abstract and intoduction sections are well written

Methods:

Statistical Analysis

1. Model Specification Issues:
a. Random Slope Specification: The phrase "random slope" is ambiguous. A random slope for which variable(s)? Typically, you would specify a random slope for a key predictor (e.g., year, or a clinic-level characteristic) if you hypothesize its effect varies by clinic. If not justified, an intercept-only model might be sufficient and more stable. Clarification is needed: Recommendation: Justify the choice of the variable(s) given random slopes. If exploring effect heterogeneity, consider model fit statistics (AIC, BIC) to compare with an intercept-only model.
b. Risk of Model Non-Convergence: Log-binomial models, especially with random effects, can suffer from convergence issues due to "non-collapsibility" and numerical instability.Recommendation: State how convergence was achieved (e.g., optimization algorithm, starting values). A robust alternative is a Poisson GLMM with a robust sandwich estimator or an over dispersed Poisson model, which is more stable and provides consistent estimates of RR, though confidence intervals may be conservative.
c. Temporal Confounding and Variable Timing: The description of client-level characteristics being summarized at three different time points (ART initiation, VL testing, first annual visit) is logical but complex. It risks introducing immortal time bias or confounding by time-updated factors if not handled meticulously. Recommendation: Provide a clear diagram or flowchart/table showing how covariates are aligned with each of the three analysis cohorts (AHD, viraemia, interruption). Consider explicitly framing these as separate, time-fixed analyses for distinct cohorts to avoid misinterpretation.

2. Variable Selection & Operationalization

a. Quintiles as Fixed Effects: Using quintiles (for clinic size, SAMPI) loses granular information and assumes a step-function relationship with the log-risk. The choice of quintiles should be justified.Recommendation: Consider using continuous variables with flexible specifications (e.g., splines) to model non-linear effects, or justify quintile categorization based on known thresholds or sample distribution.
b. "Year" as a Fixed Effect: Including year is good for temporal adjustment. However, specify its form (categorical vs. continuous). If categorical, state the reference year. If continuous, check the linearity assumption on the log-risk scale.
c. Missing Data: No mention of how missing data in covariates or outcomes is handled. Complete-case analysis can introduce bias.Recommendation: Report the amount of missingness for key variables and describe the method used (e.g., multiple imputation, which is preferred for mixed models).

3. Analysis Clarity & Reporting

a. Separate Models: It reads as if one giant model (or joint modeling for that matter) is fit for three distinct outcomes. This should be clarified.Recommendation: Explicitly state: "Separate models were fit for each outcome (AHD, treatment interruption, viraemia ≥50 copies/mL)."
b. Effect Measure Interpretation: The "clinic-level relative risk" phrasing could be misinterpreted. It's the RR associated with clinic-level characteristics (like size), not a RR per clinic.Recommendation: Rephrase to: "To estimate the association between clinic-level characteristics and the risk of [outcome], we expressed results as adjusted Relative Risks (aRR)..."

Geospatial mapping

**1. Statistical and Methodological Weaknesses**

a. Ignoring Uncertainty: This is the most critical flaw. IDW produces a single, smooth surface, giving a false impression of precision. It does not generate:
   i. Prediction Intervals: How uncertain is the estimate at each unsampled location?
   ii. Uncertainty Maps: Which areas have reliable estimates (near clinics) and which are highly speculative (far from clinics)?
   iii. Policymakers might act on "hotspots" that are, in fact, statistical artifacts of low clinic density.
b. Naïve Assumption of Spatial Structure: IDW assumes a simple, deterministic distance-decay relationship. It does not model the underlying spatial process.
   i. It ignores potential spatial autocorrelation beyond simple distance (e.g., directional trends, barriers like highways or rivers).
   ii. It cannot separate signal (true spatial pattern) from noise (random clinic-level variation).
c. Sensitivity to Parameters: The IDW output is highly sensitive to the chosen power parameter (which controls the weight of distance) and search radius. The critique is incomplete without stating the values used and their justification.*Recommendation:* The authors must report the power parameter (e.g., power=2) and the search neighborhood strategy (e.g., number of nearest clinics used).
d. Edge Effects and Extrapolation: IDW performs poorly at the edges of the study area (e.g., near provincial borders) and can produce unrealistic "bullseye" artifacts around isolated clinics. It inappropriately extrapolates into areas with no data.

**2. Inferential and Public Health Limitations**

- Cannot Adjust for Covariates: The maps show crude proportions. A high proportion in a district could be due to its demographic (e.g., older population) or clinic-level (e.g., under-resourced) characteristics, not purely geography. This leads to ecological confounding. *Recommendation:*  A geostatistical model like INLA can integrate the clinic- and client-level fixed effects from your GLMM directly into the spatial surface, producing an adjusted spatial risk map.

**Results and discussion**

Results and discussion sections could be improved with the improved approaches discussed above.
