# Supplementary material for: Trends in advanced HIV disease, treatment interruption, and viraemia in KwaZulu-Natal, South Africa
Source: PLOS Glob Public Health. 2026 Sep 2;6(9):e0005506. doi: 10.1371/journal.pgph.0005506 (PMC13537683; doi:10.1371/journal.pgph.0005506)
Supplement: Supplementary file 7 [file pgph.0005506.s017.docx]

04 June 2026

Dear Dr. Sebean Manyimbo and colleagues,

**Response to review of manuscript ID PGPH-D-25-03407**

Thank you for the feedback from the editorial team and reviewers on our manuscript entitled “Trends in advanced HIV disease, treatment interruption, and viraemia in KwaZulu-Natal, South Africa”. We are grateful for the opportunity to submit a revised version of our manuscript. Below we provide a response to each of the editor/reviewer comments. Line numbers are for the revised manuscript.

We look forward to hearing back from you.

Regards

Sanele Mbeje

**Journal Requirements:**

**Comment 1**. Please update your online Competing Interests statement. If you have no competing interests to declare, please state: "The authors have declared that no competing interests exist."

**Response:** The competing interests statement has been updated.

**Comment 2**.  We note that you have indicated that there are restrictions to data sharing for this study. For studies involving human research participant data or other sensitive data, we encourage authors to share de-identified or anonymized data. However, when data cannot be publicly shared for ethical reasons, we allow authors to make their data sets available upon request. For information on unacceptable data access restrictions, please see [http://journals.plos.org/globalpublichealth/s/data-availability#loc-unacceptable-data-access-restrictions](https://url.za.m.mimecastprotect.com/s/5_MHC48K3Ns9v183HWs7U4QSdv?domain=journals.plos.org). 

Before we proceed with your manuscript, please address the following prompts:

a) If there are ethical or legal restrictions on sharing a de-identified data set, please explain them in detail (e.g., data contain potentially identifying or sensitive patient information, data are owned by a third-party organization, etc.) and who has imposed them (e.g., a Research Ethics Committee or Institutional Review Board, etc.). Please also provide contact information for a data access committee, ethics committee, or other institutional body to which data requests may be sent.

b) If there are no restrictions, please upload the minimal anonymized data set necessary to replicate your study findings to a stable, public repository and provide us with the relevant URLs, DOIs, or accession numbers. Please see [http://www.bmj.com/content/340/bmj.c181.long](https://url.za.m.mimecastprotect.com/s/n96WC5804OspDGl4txtNUk5spB?domain=bmj.com) for guidelines on how to de-identify and prepare clinical data for publication. For a list of recommended repositories, please see [https://journals.plos.org/globalpublichealth/s/recommended-repositories](https://url.za.m.mimecastprotect.com/s/YawJC66VNPF0BKW8TLurU51-8b?domain=journals.plos.org). You also have the option of uploading the data as Supporting Information files, but we would recommend depositing data directly to a data repository if possible.

Please update your Data Availability statement in the submission form accordingly.

**Response:** We have updated the data availability statement and provided contact details in lines 378-380.

**Comment 3**. Please provide separate main figure files in .tif or .eps format only and ensure that all files are under our size limit of 10MB.

For more information about how to convert your figure files please see our guidelines: [https://journals.plos.org/globalpublichealth/s/figures](https://url.za.m.mimecastprotect.com/s/tAztC76J0QFVJkRoI0CAUoEbUt?domain=journals.plos.org)

**Response:** The main figures has been uploaded separately.

**Comment 4**. Some material included in your submission may be copyrighted. According to PLOS’s copyright policy, authors who use figures or other material (e.g., graphics, clipart, maps) from another author or copyright holder must demonstrate or obtain permission to publish this material under the Creative Commons Attribution 4.0 International (CC BY 4.0) License used by PLOS journals. Please closely review the details of PLOS’s copyright requirements here: PLOS Licenses and Copyright. If you need to request permissions from a copyright holder, you may use PLOS's Copyright Content Permission form.

Please respond directly to this email or email the journal office and provide any known details concerning your material's license terms and permissions required for reuse, even if you have not yet obtained copyright permissions or are unsure of your material's copyright compatibility.

Potential Copyright Issues:

Fig 3, Fig 5, Fig 7, S1 Fig: please

(a) provide a direct link to the base layer of the map (i.e., the country or region border shape) and ensure this is also included in the figure legend; and

(b) provide a link to the terms of use / license information for the base layer image or shapefile. We cannot publish proprietary or copyrighted maps (e.g. Google Maps, Mapquest) and the terms of use for your map base layer must be compatible with our CC-BY 4.0 license.

Note: if you created the map in a software program like R or ArcGIS, please locate and indicate the source of the basemap shapefile onto which data has been plotted.

If your map was obtained from a copyrighted source please amend the figure so that the base map used is from an openly available source. Alternatively, please provide explicit written permission from the copyright holder granting you the right to publish the material under our CC-BY 4.0 license.

Please note that the following CC BY licenses are compatible with PLOS license: CC BY 4.0, CC BY 2.0 and CC BY 3.0, meanwhile such licenses as CC BY-ND 3.0 and others are not compatible due to additional restrictions.

If you are unsure whether you can use a map or not, please do reach out and we will be able to help you. The following websites are good examples of where you can source open access or public domain maps: 
* U.S. Geological Survey (USGS) - All maps are in the public domain. ([http://www.usgs.gov](https://url.za.m.mimecastprotect.com/s/3QlTC8qYNRHPKRkLuRFvUybgc2?domain=usgs.gov)) 
* PlaniGlobe - All maps are published under a Creative Commons license so please cite “PlaniGlobe, [http://www.planiglobe.com](https://url.za.m.mimecastprotect.com/s/n9pJC98XNVsNpBO9IGHnUqLbTM?domain=planiglobe.com), CC BY 2.0” in the image credit after the caption. ([http://www.planiglobe.com/?lang=enl](https://url.za.m.mimecastprotect.com/s/q8mxC0g37JsgL3yZS9IBU9z39s?domain=planiglobe.com)) 
* Natural Earth - All maps are public domain. ([http://www.naturalearthdata.com/about/terms-of-use/](https://url.za.m.mimecastprotect.com/s/uzytCg5XElIw72BpCJS7U4aBPu?domain=naturalearthdata.com/))

**Response:** We have updated the captions of all the figures (Figs 3, 5, 7 and S1 Fig) to credit the map source and provided direct links to the source. We obtained the base layer of the maps from an open-access portal of the South African Municipal Demarcation Board.

Direct link to ward boundaries [MDB Wards 2020 | Municipal Demarcation Board](https://dataportal-mdb-sa.opendata.arcgis.com/datasets/279fbf82a48f46678ddd498627af3f0a_0/explore?location=-28.479600%2C24.698437%2C6)

Direct link to local municipality boundaries [MDB Local Municipal Boundary 2018 | Municipal Demarcation Board](https://dataportal-mdb-sa.opendata.arcgis.com/datasets/27bbdd5b041b4ba6b5707dfed5aa3923_0/explore?location=-28.439167%2C24.609789%2C6)

**Comment 5**. If the reviewer comments include a recommendation to cite specific previously published works, please review and evaluate these publications to determine whether they are relevant and should be cited. There is no requirement to cite these works unless the editor has indicated otherwise.

**Response:** Done

**Comment 6**. Please review your reference list to ensure that it is complete and correct. If you have cited papers that have been retracted, please include the rationale for doing so in the manuscript text, or remove these references and replace them with relevant current references. Any changes to the reference list should be mentioned in the rebuttal letter that accompanies your revised manuscript. If you need to cite a retracted article, indicate the article’s retracted status in the References list and also include a citation and full reference for the retraction notice.

**Response:** Done

**Reviewers’ Comments to Author:**

**Reviewer #1:**

**Comment**: Abstract - line 38 - here, and elsewhere in the manuscript - consider using the term "unplanned treatment interruption" - to distinguish it from Structured Treatment Interruptions, which might come to mind for readers who are clinicians. Alternatively, the distinction can be made in the paragraph 123 to 127, where definitions are described.

**Response:** We thank the reviewer for highlighting this important aspect of the definition of interruption. We have provided a clear definition of treatment interruption in the Outcomes section (lines 128-129), the new definition now reads as: “We defined treatment interruption as an unplanned break in HIV care, measured by whether a PLWH missed any scheduled visit by more than 90 days”.

**Comment**: line 49 - could a p-value be given to give a sense of whether this reduction of treatment interruption is significant?

**Response:** We have rephrased this sentence (lines 47-48) as follows: “The prevalence of treatment interruptions ranged between 11.3% and 13.2% among the 544,066 clients who had scheduled visits”. The decline was not significant, please refer to p-values provided in Table 2 from the model which assesses how these outcomes have changed over the years in clinics.

**Comment**: Lines 91 to 92 - this data on viraemia looks very similar to yours - what distinguishes your study from this one?

**Response:** The cited manuscript uses NHLS data to describe the proportion of viraemic samples in KZN. We focused on clients with sustained ART exposure (>5 months) selecting the worst VL per client per year, and performed a more localized, granular analysis of the KZN region. We used TIER.Net data, which allowed us access to additional variables not available in NHLS. Thus, our study also includes additional outcomes of advanced HIV disease and treatment interruption.

**Comment**: line 102 to 103 - Describe the districts here e.g. rural and urban composition of each. Why have you chosen these three districts in particular?
And the reason for mentioning one hospital within one of the districts is unclear to the reader.

## **Response**: We have added a supplementary figure (S1 Fig) which describes the level of urbanization. We did not select these districts or clinics, these were the clinics for which we had data access at the time of the analysis. For uMkhanyakude, we only had access to the clinics managed by Mseleni hospital and Bethesda hospital which is why it was mentioned. We have added details related to this in the Study design, setting and population section (lines 103-105): “We conducted a retrospective cohort study of clients from 116 primary healthcare clinics in KwaZulu-Natal for which we had data. These clinics are managed by the eThekwini Municipality, uMgungudlovu District, and Mseleni Hospital and Bethesda Hospital in uMkhanyakude District.”

**Comment**: line 124 - I would reference/justify the definition of treatment interruption (>90d) - especially given the discussion section, where you talk about the heterogeneity of definitions and thus findings.

**Response:** We have revised and added references for the definition of treatment interruption under the outcome section (lines128-129). “We defined treatment interruption as an unplanned break in HIV care, measured by whether a PLWH missed any scheduled visit by more than 90 days”

- Arnesen, R., A.P. Moll, and S.V. Shenoi, *Predictors of loss to follow-up among patients on ART at a rural hospital in KwaZulu-Natal, South Africa.* PLoS One, 2017. **12**(5): p. e0177168.
- Fatti, G., A. Grimwood, and P. Bock, *Better antiretroviral therapy outcomes at primary healthcare facilities: an evaluation of three tiers of ART services in four South African provinces.* PLoS One, 2010. **5**(9): p. e12888
- Chauke, P., M. Huma, and S. Madiba, *Lost to follow up rate in the first year of ART in adults initiated in a universal test and treat programme: a retrospective cohort study in Ekurhuleni District, South Africa.* Pan Afr Med J, 2020. **37**: p. 198.
- South African National Department of Health, *2023 ART clinical guidelines for the management of HIV in adults, pregnancy and breastfeeding, adolescents, children, infants and neonates.* South Africa, 2023.

**Comment**: Table 2 - the relationship between "standardized median age" and RR for AHD or viraemia is unclear to the reader - is it: the higher the median age, the greater the risk of AHD and the lower the risk of viraemia?

**Response:** The reviewer’s interpretation is correct: a higher clinic median age is associated with an increased risk of AHD and reduced risk of viraemia. We have clarified that age was standardized using Z - scores, where a one-unit change implies a one standard deviation change using a footnote in Table 2 (lines 220-223) and revised the interpretation (lines 214-215): “For every one standard deviation (SD) increase (1.84 years) in the median age of clients in a clinic, the risk of AHD increased by 7% (RR: 1.07, 95%CI: 1.04-1.09)”. and lines 271-272 “Clinics serving older clients (1.57 years increase in median age) had a reduced risk of viraemia (RR: 0.92, 95% CI: 0.91-0.94)”

**Comment**: line 286 - It would have made more sense to correlate the client residential location with poverty index, rather than clinic ward - clinics drain clients from the surrounding area and neighbouring areas, which might have different levels of poverty.

**Response:** We acknowledge that clients residential location data would provide a more precise measure of the poverty index. However, we have no access to client’s residential locations due to confidentiality and data privacy protocols. Hence, clinic’s ward serves as proxy for the broader socio-economic status. We acknowledged this in the limitations (lines 351-353), “First, the analysis relied on clinic coordinates rather than client residential area. Clients residential location could have supported a more accurate estimation of clinic catchment areas and allowed us to assess spatial autocorrelation beyond a distance (such as directional trends or physical geographic barriers)”

**Reviewer #2**

**Comment:** A general comment, the authors may consider including the rationale for the selection of facilities and districts and not including all health districts in KwaZulu-Natal. Whilst this does not impact on the generalisability of the results, it certainly would contextualise the study for the reader.

**Response:** We currently only have permission to access the data for the districts considered in the study, and in two of these districts, eThekwini and uMkhanyakude, we only have access to some of the clinics’ data; in eThekwini, we only have access to municipality clinics and only clinics managed by Mseleni hospital and Bethesda hospital in uMkhanyakude. We have added detail related to this in the Study design, setting and population section (lines 103-105): “We conducted a retrospective cohort study of clients from 116 primary healthcare clinics in KwaZulu-Natal for which we had data. These clinics are managed by the eThekwini Municipality, uMgungudlovu District, and Mseleni Hospital and Bethesda Hospital in uMkhanyakude District.”

**Comment:** Line 222-223: This sentence may need clarification: the interpretation of the reduced risk of viraemia in older clients, is it risk of viraemia (≥ 50copies/ml)? Authors need to be consistent with this term. While viraemia is defined as a detectable HIV viral load in plasma, further classification into low-level viraemia (≥ 50 - < 100 copies/ml) and high-level viraemia (≥ 1000 copies/ml) would improve interpretation of results and has programmatic implications. A further suggestion, the authors might consider classifying adjusted relative risk by HIV viral load thresholds (low-level versus high-level viraemia), this distinction will improve interpretation and application of these study findings. An increase in low-level viraemia(≥50 -<1000 copies/ml) is concerning as persistent HIV viral loads between 200 and 500 copies/ml is predictive of future virological failure and viral loads between 400 and 999 copies/ml are associated with increased 10-year risk of mortality. Distinguishing the two have implications for public health interventions.

Line 270- 271: Authors need to distinguish between low-level and high-level viraemia. General note, authors need to be consistent when classifying viraemia as “high-level viraemia” versus viraemia (≥1000 copies/ml) and “low-level viraemia” versus viraemia (≥ 50 copies/ml).

**Response:** We thank the reviewer for this clinical observation on viral load thresholds. As mentioned in the statistical analysis section lines 150-152, we assessed the association between clinic level characteristics and viraemia (≥50 copies/mL), hence all the interpretation of the model results related to viraemia refers to ≥50 copies/mL. While we acknowledge that distinction between low-level viraemia (50-999 copies/mL) and high level viraemia (≥1000 copies/mL) would provide additional granularity, our primary objective was to assess the broad programmatic threshold of viraemia (≥50 copies/mL). We have clarified this in the section called outcomes (lines 135-137): “Although our main outcome of viraemia was defined using a threshold of 50 copies/mL, in some analyses we additionally report results using VL ≥1000 copies/mL in a year.”

The trends for low level viraemia (50<= VL<1000 copies/mL) are also provided in supplementary information, overall and at a district level (S6-7 Figs)

**Comment:** Line 290-292: Whilst the conclusion includes the key findings of the study and emphasises the need for more targeted programmatic interventions, the authors are encouraged to expand on the utility of geospatial analysis in a local context. Spatiotemporal analysis has the advantage of being used as near real-time monitoring and surveillance tool for informing targeted and tailored public health responses.

**Response:** We have revised the conclusion (lines 365-370) as follows: “Crucially, our findings also revealed geographic heterogeneity in these measures. The persistent burden of AHD, increasing viraemia, and marked spatial variation across the studied districts underscores the need for continued geospatial analysis. By uncovering these localized disparities, this study highlights the unique utility of geospatial analysis as a tool for near real-time epidemiology surveillance, providing the empirical foundation to design geographically tailored interventions to strengthen programmatic effectiveness in hyper-endemic settings like KwaZulu-Natal.”

**Reviewer #3:** 

Statistical Analysis
1. Model Specification Issues:
**Comment:** a. Random Slope Specification: The phrase "random slope" is ambiguous. A random slope for which variable(s)? Typically, you would specify a random slope for a key predictor (e.g., year, or a clinic-level characteristic) if you hypothesize its effect varies by clinic. If not justified, an intercept-only model might be sufficient and more stable. Clarification is needed: Recommendation: Justify the choice of the variable(s) given random slopes. If exploring effect heterogeneity, consider model fit statistics (AIC, BIC) to compare with an intercept-only model.

**Response**: We have updated the manuscript under statistical analysis section to explicitly state that year was specified as a random slope at the clinic level. The choice of the random slope was justified based on the clinical justification that individual clinics would exhibit heterogeneity in their progress. Added text (lines 156-158): “Each model also included clinic-specific random effects on the intercept to account for the heterogeneity in outcomes across clinics and on year to allow the longitudinal trajectory of the outcomes to vary by clinics.”

**Comment:** b. Risk of Model Non-Convergence: Log-binomial models, especially with random effects, can suffer from convergence issues due to "non-collapsibility" and numerical instability. Recommendation: State how convergence was achieved (e.g., optimization algorithm, starting values). A robust alternative is a Poisson GLMM with a robust sandwich estimator or an over dispersed Poisson model, which is more stable and provides consistent estimates of RR, though confidence intervals may be conservative.

**Response:** We acknowledge the reviewer's concern regarding numerical instability and potential for non-convergence in log-binomial mixed models. To ensure stability and validity of our estimates across all three models, parameters were estimated via Maximum Likelihood using the Laplace Approximation within glmer function from the lme4 package in R. To address the risk of non-convergence, we used the bobyqa optimizer with default settings. Additionally, we applied data-scaling techniques to reduce eigenvalue disparities and optimization, the median age was standardized using Z-scores, the proportion of male clients was mean-centered, and both clinic size and multidimensional poverty index were modelled as quintiles.

All models successfully converged without any optimization warnings or indications of singular fits. Because convergence was fully achieved alternative approach such Poisson GLMM with robust sandwich estimators was not required, allowing us to keep the log-binomial models without risking conservative confidence intervals.

We have provided details on how the models were fit in the supplementary information alongside supplementary tables under model estimation.

**Comment:** c. Temporal Confounding and Variable Timing: The description of client-level characteristics being summarized at three different time points (ART initiation, VL testing, first annual visit) is logical but complex. It risks introducing immortal time bias or confounding by time-updated factors if not handled meticulously. Recommendation: Provide a clear diagram or flowchart/table showing how covariates are aligned with each of the three analysis cohorts (AHD, viraemia, interruption). Consider explicitly framing these as separate, time-fixed analyses for distinct cohorts to avoid misinterpretation.

**Response**: We thank the reviewer for this critical feedback. We curated different analysis populations, as shown in the flow diagram (Fig 1)

For AHD analysis: We summarised characteristics of clients at the time of initiation (cross-sectional at entry) as shown in supplementary information (S1 Table). Characteristics (age, sex, district) and the outcome (CD4 count/AHD status) were measured simultaneously at entry, and since there is no follow-up period between the outcome and measurements, immortal time bias is excluded.

Viraemia and interruption: We treated these as a series of annual cross-sectional snapshots of the active population. Age was recalculated for each year (at the time of viral load testing/ first visit in each year) and this accounts for time updated factors (age). Note that all these characteristics were summarised at a clinic level before being used for analysis. Please refer to S2 and S3 Tables in the supplementary information.

2. Variable Selection & Operationalization
**Comment:** a. Quintiles as Fixed Effects: Using quintiles (for clinic size, SAMPI) loses granular information and assumes a step-function relationship with the log-risk. The choice of quintiles should be justified. Recommendation: Consider using continuous variables with flexible specifications (e.g., splines) to model non-linear effects, or justify quintile categorization based on known thresholds or sample distribution.

**Response:** We thank the reviewer for this insightful comment regarding the choice of functional form of SAMPI and clinic size. Our justification for modelling SAMPI variable using quintiles was taken from the literature as referenced in the methods section (line 146-149). “Madela, S., et al., *Individual and area-level socioeconomic correlates of hypertension prevalence, awareness, treatment, and control in uMgungundlovu, KwaZulu-Natal, South Africa.* BMC Public Health, 2023. **23**(1): p. 417.” and to also facilitate model convergence as described earlier.

Regarding the clinic size, there are no clear guidelines or known administrative thresholds to define clinic sizes (e.g. small versus large), as this often depends on the specific services offered by the facility. We therefore utilized sample distribution-based quintiles. This approach allowed us to assess variation across different clinic sizes while also facilitating model convergence and to deal with the eigenvalue issues.

**Comment:** b. "Year" as a Fixed Effect: Including year is good for temporal adjustment. However, specify its form (categorical vs. continuous). If categorical, state the reference year. If continuous, check the linearity assumption on the log-risk scale.

**Response:** We appreciate the reviewer’s comment on this important consideration. We have updated the methods section to specify that year was included as a continuous variable (line 154-158).

“Each outcome model included fixed effects for clinic median age, clinic size quintile, clinic SAMPI quintile, clinic proportion of males and a continuous variable for year of ART initiation / scheduled visit / VL testing. Each model also included clinic-specific random effects on the intercept to account for the heterogeneity in outcomes across clinics and on year to allow the longitudinal trajectory of the outcomes to vary by clinics”.

While we have explored alternative functional forms to test the linearity assumption on log-risk scale using second-order polynomial and cubic splines, we retained the linear form for two main reasons. First, our primary research objective was to assess the direction of the trend in these outcomes over the study period, and secondly, the length of the longitudinal period is short, for each clinic there are only 5 annual data points. The inclusion of random slopes for the year, however, helps to account for heterogeneity in longitudinal trajectories across different clinics.

**Comment:** c. Missing Data: No mention of how missing data in covariates or outcomes is handled. Complete-case analysis can introduce bias. Recommendation: Report the amount of missingness for key variables and describe the method used (e.g., multiple imputation, which is preferred for mixed models).

**Response**: We thank the reviewer for highlighting the importance of addressing potential bias from missing data. In this study, our analysis was conducted using aggregated clinic-level data rather than individual client-level records. Consequently, the dataset used for the models (comprising 580 observations across 116 facilities) contained no missing data for the outcomes or the covariates (year, gender distribution, clinic size and multidimensional poverty index). At a client level, only the age variable had missing values, and the prevalence of missing data was very small (<5%).

3. Analysis Clarity & Reporting
**Comment:** a. Separate Models: It reads as if one giant model (or joint modeling for that matter) is fit for three distinct outcomes. This should be clarified. Recommendation: Explicitly state: "Separate models were fit for each outcome (AHD, treatment interruption, viraemia ≥50 copies/mL)."

**Response:** We have updated the manuscript to explicitly state that separate models were fitted (lines 150-153), we have rephrased the statistical analysis section as “To assess the association between clinic-level characteristics and each outcome (AHD, treatment interruption and viraemia (≥50 copies/mL), we used separate log-binomial generalized linear mixed models (GLMM) for each outcome, with results expressed as adjusted relative risk (aRR) with 95% confidence intervals (CIs).”

**Comment:** b. Effect Measure Interpretation: The "clinic-level relative risk" phrasing could be misinterpreted. It's the RR associated with clinic-level characteristics (like size), not a RR per clinic. Recommendation: Rephrase to: "To estimate the association between clinic-level characteristics and the risk of [outcome], we expressed results as adjusted Relative Risks (aRR)..."

**Response**: Done, please see the previous response.

Geospatial mapping

1. Statistical and Methodological Weaknesses
a. Ignoring Uncertainty: This is the most critical flaw. IDW produces a single, smooth surface, giving a false impression of precision. It does not generate:
i. Prediction Intervals: How uncertain is the estimate at each unsampled location?
ii. Uncertainty Maps: Which areas have reliable estimates (near clinics) and which are highly speculative (far from clinics)?
iii. Policymakers might act on "hotspots" that are, in fact, statistical artifacts of low clinic density.

**Response:** We thank the reviewer for highlighting this important limitation. We agree that the deterministic interpolation method, IDW, does not demonstrate uncertainty of the estimates. Our intention with these maps was strictly descriptive and exploratory, to visualize the crude spatial distribution of our outcomes. We have included indicators for the location of clinics on the maps (Figs 3, 5, and 7) so that it is clear which areas have prevalence estimates based on far-lying clinics and thus are susceptible to greater uncertainty. We acknowledged the weaknesses of the method used in the discussion section (lines 355-358): “Secondly, the use of the IDW method introduces specific limitations. In particular, the underlying algorithm relies on the distance between each sampled clinic and the unsampled point, it does not adjust for covariates, lacks the capacity to generate prediction intervals to quantify estimation uncertainty, and fails to account for directional trends.”

b. Naïve Assumption of Spatial Structure: IDW assumes a simple, deterministic distance-decay relationship. It does not model the underlying spatial process.
i. It ignores potential spatial autocorrelation beyond simple distance (e.g., directional trends, barriers like highways or rivers).
ii. It cannot separate signal (true spatial pattern) from noise (random clinic-level variation).

**Response:** We acknowledge that the IDW relies on a deterministic mathematical distance decay function rather than modelling the spatial autocorrelation. However, we opted for IDW to avoid making complex, unjustifiable assumptions about the spatial structure given our data constraints. Due to strict client confidentiality and data privacy regulations, client residential locations were not available. Having access to such granular data would be necessary to robustly to assess advanced spatial autocorrelation beyond using a distance. We have clarified the descriptive intent of mapping of the maps and acknowledged these limitations in the discussion (lines 349-358). “Geospatial mapping was used as a descriptive tool to visualize observed spatial burdens at across participating clinics. However, these findings should be interpreted with caution due to several challenges. First, the analysis relied on clinic coordinates rather than client residential area. Clients residential location could have supported a more accurate estimation of clinic catchment areas and allowed us to assess spatial autocorrelation beyond a distance (such as directional trends or physical geographic barriers). Nonetheless, the use of clinic locations remains valuable, by identifying where healthcare system experiences the highest burden. Secondly, the use of the IDW method introduces specific limitations. In particular, the underlying algorithm relies on the distance between each sampled clinic and the unsampled point, it does not adjust for covariates, lacks the capacity to generate prediction intervals to quantify estimation uncertainty, and fails to account for directional trends”

c. Sensitivity to Parameters: The IDW output is highly sensitive to the chosen power parameter (which controls the weight of distance) and search radius. The critique is incomplete without stating the values used and their justification. Recommendation: The authors must report the power parameter (e.g., power=2) and the search neighborhood strategy (e.g., number of nearest clinics used).

**Response:** We agree with the reviewer on the importance of this omission. We have updated the methods section (lines 172-174) to explicitly state our parameters “The algorithm was configured with a default distance coefficient (power parameter) of 2. To preserve the influence of proximate clinics and prioritize local variation, we restricted to a maximum of 3 nearest neighbors, with the assumption that clients are most likely to seek care at clinics within their immediate geographic vicinity.”

d. Edge Effects and Extrapolation: IDW performs poorly at the edges of the study area (e.g., near provincial borders) and can produce unrealistic "bullseye" artifacts around isolated clinics. It inappropriately extrapolates into areas with no data.

**Response:** We thank the reviewer for raising these important points regarding the IDW limitations. We have added indicators of all analysed clinics onto the maps. This allows readers to directly evaluate data density and identify isolated clinics, where uncertainty and interpolation artifacts are more likely to occur. We acknowledged the weaknesses in the discussion line 358-362, “The method is also prone to the “bull’s eye” effect around isolated sampled points, where the measured point only influences a small area around it, which does not reflect actual geographic transition. All maps (Figs 3, 5 and 7) are overlayed with locations of the clinics used in the analysis to assist readers in identifying isolated clinics where uncertainty and interpolation artifacts are more likely to occur.”

2. Inferential and Public Health Limitations
• Cannot Adjust for Covariates: The maps show crude proportions. A high proportion in a district could be due to its demographic (e.g., older population) or clinic-level (e.g., under-resourced) characteristics, not purely geography. This leads to ecological confounding. Recommendation: A geostatistical model like INLA can integrate the clinic- and client-level fixed effects from your GLMM directly into the spatial surface, producing an adjusted spatial risk map.

**Response:** We thank the reviewer for the excellent point about difference between crude patterns and adjusted spatial risk. We agree that the IDW does not adjust for clinic or client-level covariates. However, the current descriptive maps serve our immediate objective of highlighting geographic areas with clinics with high burden. We acknowledged the weaknesses in the discussion section (lines 355-358) “Secondly, the use of the IDW method introduces specific limitations. In particular, the underlying algorithm relies on the distance between each sampled clinic and the unsampled point, it does not adjust for covariates, lacks the capacity to generate prediction intervals to quantify estimation uncertainty, and fails to account for directional trends”

**Reviewer #4:**

**Comment:** Line 47-52: What was the total number of clients for the study? It is noted that 123,473 initiating ART had CD4 count tests done. May you also indicate the number of clients initiating ART who do not have their CD4 count tests? ( This will be helpful in addressing the gap of missed opportunities for AHD diagnosis and management). Also recommending an indication of clients who had their VL due but were not done.

**Response**: We thank the reviewer for this comment. The total number of clients is given in the results section (line 193) “The total number of clients who were on ART and included in this study was 551,557 (Fig 1A).” A flow diagram (Fig 1) was also updated to show the number of clients with and without CD4 count. We have included details on the proportion of clients who were missing VL results in the results (line 251-252): “The proportion of clients who had VL results was 76.9% in July2018–June2019 and increased to 82.7% in July2022–June2023 (Fig 6).”

Introduction

**Comment:** Line 71-75: May you also include a reference to the South African Guidelines regarding management of ART-AHD.

**Response:** We have updated the manuscript and added the reference (lines 70-75) as “Following the 2017 WHO recommendations for a comprehensive AHD care package, the 2019 SA ART guidelines operationalized a standard package, prioritizing rapid ART initiation and co-infection prophylaxis alongside intensified screening for opportunistic infections. Specifically, the guidelines mandate reflex laboratory screening for cryptococcal antigen (CrAg) for all clients with CD4<100 cells/mm^3^, and the use of lateral flow urine lipoarabinomannan (U-LAM) for rapid tuberculosis screening”

**Comment:** Line 93-95: Is there evidence for other provinces? Recommendation to include evidence for other provinces (from the 9 provinces, is the evidence only from Mpumalanga?)

**Response**: We have updated the manuscript (lines 93-95) as follows “Viral suppression rates also vary by province; as of 2022, Limpopo had the lowest prevalence of viral suppression (<1000 copies/mL) among adults aged 15+ years (75.3%), and KwaZulu-Natal had the highest (86.7%)”.

These findings were reported by HSRC “The Sixth South African National HIV Prevalence, Incidence, and Behaviour Survey (SABSSM VI): Summary sheet October 2023” available from https://sahivsoc.org/Files/SABSSMVI-SUMMARY-SHEET-2023.pdf

**Comment:** Line 109-110: It is necessary to include the Viral load testing for viremia (management of viremia) as per the SA guidelines.

**Response**: We have updated the manuscript (lines 110-114) as follows “As per South African 2019 guidelines, VL testing was recommended to be done at 6 months on ART, at 12 months if virally suppressed (<50 copies/mL) and annually thereafter for those who remained virally suppressed. For clients with VL ≥50 copies/mL, the guidelines recommended that the elevated VLs should be managed with enhanced adherence counselling and repeat of VL testing within 3 months. Meanwhile, CD4 count testing is recommended at initiation, and when clinically indicated”

Results
**Comment:** Line 171-173: What was the total number of clients for the study? And disaggregation according to males and females

**Response**: The number of clients who were on ART during study periods was 551,557 (line 193), please refer to the flow diagram (Fig 1) showing how the 3 analysis populations were achieved. The number clients in terms of sex are summarised in S1-3 Tables in supplementary information.

**Comment:** Line 177-178: Clarify the period for ART initiation (2018-2023) for the clients included in the AHD analysis.

**Response**: Since implementation on universal treatment in 2016 which was also adopted by SA later in the year, by 2018 this policy was fully integrated into primary healthcare clinics. Data from 2018 onwards represent a stabilised period where eligibility for ART was no longer tied to a CD4 count.

**Comment:** Line 184-186: Figure 1- Flow chart for each study Outcome
A: Please add the number of clients with CD4>200 and CD4<200
C: Please add the number of clients with VL<50, VL>50-999 and VL>1000
These will help expand understanding of the burden of AHD, including viremia.
It would also be appreciated to disaggregate these outcomes by gender

**Response**: We have updated the flow chart (Fig 1) to indicate the number of clients with CD4 counts>=200 and <200 cells/mm^3^. For the viral load data, we have indicated the number of VLs <50, 50-999 and >=1000 copies/mL not the number of clients. This is because clients were followed up longitudinally and therefore, they can be counted more than once.

**Comment:** Line 194-196: What were the findings for females?

**Response:** We appreciate the reviewer’s inquiry regarding the findings for females. Because our data was aggregated at a clinic-level, the proportion of males and females are perfectly collinear, including both variables would introduce redundancy, making the model unstable. Findings for males implies the inverse for females.

Discussion
**Comment:** Line 243, 261, 274: What are the attributions for the geographical distributions (rural and urban) of AHD, Treatment Interruptions and Viremia? Also, what are the attributions for the gender disparities?
What were the observations regarding the management of viral load and viremia? (Line 211-212 showed that 76.9% of clients had VL results.) Also, the attributions for viremia in line 217 - geographical distribution.

**Response:** We thank the reviewer for highlighting these important considerations. We revised the discussion section (lines 331-339) as follows “The results from our spatial analysis revealed that the burdens of AHD, treatment interruptions, and viraemia (≥50 copies/mL) varies across and within the studied districts, spanning urban, peri-urban and rural areas. In rural communities such as parts of uMgungundlovu and uMkhanyakude districts, these challenges may be attributed to disparate access to HIV testing delaying early access to ART, and declines in ART initiations during COVID-19 pandemic. Structural barriers such as geographic isolation, supply stockouts and long travel distances to clinics may also contribute. However, in high-density urban settings within eThekwini and uMgungundlovu, these challenges may be driven by higher prevalence of HIV, clinic congestion, informal settlement dynamics, and high levels of migration for employment which disrupt care continuity.” However, due to constraints regarding clinic characteristics and clients health seeking behaviors, the current study was unable to determine or assess reasons for the observed variation across different regions.
