# Supplementary material for: Trends in advanced HIV disease, treatment interruption, and viraemia in KwaZulu-Natal, South Africa
Source: PLOS Glob Public Health. 2026 Sep 2;6(9):e0005506. doi: 10.1371/journal.pgph.0005506 (PMC13537683; doi:10.1371/journal.pgph.0005506)
Supplement: Peer review history

# Peer review: Decision Letter 0

**DOI:** 10.1371/journal.pgph.0005506.r001

**Authors:** Sebean Mayimbo

26 Feb 2026

PGPH-D-25-03407

Trends in advanced HIV disease, treatment interruption, and viraemia in KwaZulu-Natal, South Africa.

PLOS Global Public Health

Dear Dr. Jienchi Dorward,

Thank you for submitting your manuscript to PLOS Global Public Health. After careful consideration, we feel that it has merit but does not fully meet PLOS Global Public Health’s publication criteria as it currently stands. Therefore, we invite you to submit a revised version of the manuscript that addresses the points raised during the review process.

Please submit your revised manuscript by 25th March, 2026. If you will need more time than this to complete your revisions, please reply to this message or contact the journal office at globalpubhealth@plos.org. When you're ready to submit your revision, log on to https://www.editorialmanager.com/pgph/ and select the 'Submissions Needing Revision' folder to locate your manuscript file.

Please include the following items when submitting your revised manuscript:

- A letter that responds to each point raised by the editor and reviewer(s). You should upload this letter as a separate file labeled 'Response to Reviewers'.
- A marked-up copy of your manuscript that highlights changes made to the original version. You should upload this as a separate file labeled 'Revised Manuscript with Track Changes'.
- An unmarked version of your revised paper without tracked changes. You should upload this as a separate file labeled 'Manuscript'.

Guidelines for resubmitting your figure files are available below the reviewer comments at the end of this letter.

We look forward to receiving your revised manuscript.

Kind regards,

Sebean Mayimbo, PhD

Academic Editor

PLOS Global Public Health

Journal Requirements:

1. Please update your online Competing Interests statement. If you have no competing interests to declare, please state: "The authors have declared that no competing interests exist."

2.  We note that you have indicated that there are restrictions to data sharing for this study. For studies involving human research participant data or other sensitive data, we encourage authors to share de-identified or anonymized data. However, when data cannot be publicly shared for ethical reasons, we allow authors to make their data sets available upon request. For information on unacceptable data access restrictions, please see http://journals.plos.org/globalpublichealth/s/data-availability#loc-unacceptable-data-access-restrictions.

Before we proceed with your manuscript, please address the following prompts:

a) If there are ethical or legal restrictions on sharing a de-identified data set, please explain them in detail (e.g., data contain potentially identifying or sensitive patient information, data are owned by a third-party organization, etc.) and who has imposed them (e.g., a Research Ethics Committee or Institutional Review Board, etc.). Please also provide contact information for a data access committee, ethics committee, or other institutional body to which data requests may be sent.

b) If there are no restrictions, please upload the minimal anonymized data set necessary to replicate your study findings to a stable, public repository and provide us with the relevant URLs, DOIs, or accession numbers. Please see http://www.bmj.com/content/340/bmj.c181.long for guidelines on how to de-identify and prepare clinical data for publication. For a list of recommended repositories, please see https://journals.plos.org/globalpublichealth/s/recommended-repositories. You also have the option of uploading the data as Supporting Information files, but we would recommend depositing data directly to a data repository if possible.

Please update your Data Availability statement in the submission form accordingly.

3. Please provide separate main figure files in .tif or .eps format only and ensure that all files are under our size limit of 10MB.

For more information about how to convert your figure files please see our guidelines: https://journals.plos.org/globalpublichealth/s/figures

4. Some material included in your submission may be copyrighted. According to PLOS’s copyright policy, authors who use figures or other material (e.g., graphics, clipart, maps) from another author or copyright holder must demonstrate or obtain permission to publish this material under the Creative Commons Attribution 4.0 International (CC BY 4.0) License used by PLOS journals. Please closely review the details of PLOS’s copyright requirements here: PLOS Licenses and Copyright. If you need to request permissions from a copyright holder, you may use PLOS's Copyright Content Permission form.

Please respond directly to this email or email the journal office and provide any known details concerning your material's license terms and permissions required for reuse, even if you have not yet obtained copyright permissions or are unsure of your material's copyright compatibility.

Potential Copyright Issues:

Fig 3, Fig 5, Fig 7, S1 Fig: please (a) provide a direct link to the base layer of the map (i.e., the country or region border shape) and ensure this is also included in the figure legend; and (b) provide a link to the terms of use / license information for the base layer image or shapefile. We cannot publish proprietary or copyrighted maps (e.g. Google Maps, Mapquest) and the terms of use for your map base layer must be compatible with our CC-BY 4.0 license.

Note: if you created the map in a software program like R or ArcGIS, please locate and indicate the source of the basemap shapefile onto which data has been plotted.

If your map was obtained from a copyrighted source please amend the figure so that the base map used is from an openly available source. Alternatively, please provide explicit written permission from the copyright holder granting you the right to publish the material under our CC-BY 4.0 license.

Please note that the following CC BY licenses are compatible with PLOS license: CC BY 4.0, CC BY 2.0 and CC BY 3.0, meanwhile such licenses as CC BY-ND 3.0 and others are not compatible due to additional restrictions.

If you are unsure whether you can use a map or not, please do reach out and we will be able to help you. The following websites are good examples of where you can source open access or public domain maps:

* U.S. Geological Survey (USGS) - All maps are in the public domain. (http://www.usgs.gov)

* PlaniGlobe - All maps are published under a Creative Commons license so please cite “PlaniGlobe, http://www.planiglobe.com, CC BY 2.0” in the image credit after the caption. (http://www.planiglobe.com/?lang=enl)

* Natural Earth - All maps are public domain. (http://www.naturalearthdata.com/about/terms-of-use/)

5. If the reviewer comments include a recommendation to cite specific previously published works, please review and evaluate these publications to determine whether they are relevant and should be cited. There is no requirement to cite these works unless the editor has indicated otherwise.

6. Please review your reference list to ensure that it is complete and correct. If you have cited papers that have been retracted, please include the rationale for doing so in the manuscript text, or remove these references and replace them with relevant current references. Any changes to the reference list should be mentioned in the rebuttal letter that accompanies your revised manuscript. If you need to cite a retracted article, indicate the article’s retracted status in the References list and also include a citation and full reference for the retraction notice.

Additional Editor Comments (if provided):

The manuscript covers an important aspect on Trends in Advanced HIV disease. The methods utilized are clear although there might be need to revisit the concerns of the third reviewer on statistical analysis. There would be need to seek authority to access the data utilized. Since there is anonymity of the patients depicted in the picture then there is confidentiality.

[Note: HTML markup is below. Please do not edit.]

Reviewers' comments:

Reviewer's Responses to Questions

**Comments to the Author**

1. Does this manuscript meet PLOS Global Public Health’s publication criteria?

Reviewer #1: Yes

Reviewer #2: Yes

Reviewer #3: Partly

Reviewer #4: Yes

**********

2. Has the statistical analysis been performed appropriately and rigorously?

Reviewer #1: Yes

Reviewer #2: Yes

Reviewer #3: No

Reviewer #4: Yes

**********

3. Have the authors made all data underlying the findings in their manuscript fully available (please refer to the Data Availability Statement at the start of the manuscript PDF file)?

The PLOS Data policy

Reviewer #1: Yes

Reviewer #2: Yes

Reviewer #3: Yes

Reviewer #4: No

**********

4. Is the manuscript presented in an intelligible fashion and written in standard English?

Reviewer #1: Yes

Reviewer #2: Yes

Reviewer #3: Yes

Reviewer #4: Yes

**********

Reviewer #1: Thank you for this well-written manuscript, on using high-level data to describe and target programme effectiveness.

Minor, specific commnets:

Abstract - line 38 - here, and elsewhere in the manuscript - consider using the term "unplanned treatment interruption" - to distinguish it from Structured Treatment Interruptions, which might come to mind for readers who are clinicians. Alternatively, the distinction can be made in the paragraph 123 to 127, where definitions are described.

line 49 - could a p-value be given to give a sense of whether this reduction of treatment interruption is significant?

Lines 91 to 92 - this data on viraemia looks very similar to yours - what distinguishes your study from this one?

line 102 to 103 - Describe the districts here e.g. rural and urban composition of each. Why have you chosen these three districts in particular?

And the reason for mentioning one hospital within one of the districts is unclear to the reader.

line 124 - I would reference/justify the definition of treatment interruption (>90d) - especially giventhe discussion section ,where you talk about the heterogeneity of definitions and thus findings.

Table 2 - the relationship between "standardized median age" and RR for AHD or viraemia is unclear to the reader - is it: the higher the median age, the greater the risk of AHD and the lower the risk of viraemia?

line 286 - It would have made more sense to correlate the client residential location with poverty index, rather than clinic ward - clinics drain clients from the surrrounding area and neighbouring areas, which might have diffierent levels of poverty.

Overall comment:

there was little exploration of the potential reasons for geographic heterogeneity - what are the identifiable features of the hotspots you have identified? Was there any overlap between the three hotspot maps, indicating common risks that might exist for allyour measured outcomes, in those areas?

Reviewer #2: Reviewer report PGPH-D-25-03407

The research article manuscript on “Trends in advanced HIV disease, treatment interruption, and viraemia in KwaZulu-Natal, South Africa” by Jienchi Dorward et al provides important insights into the geographical disparities of the HIV care continuum down to a facility level in KwaZulu-Natal, a high prevalence HIV setting. The authors present trends on advanced HIV disease (AHD), treatment interruption and viraemia in a retrospective cohort study using data from a national HIV electronic register. The authors further used interpolation methods to illustrate the differences in programme performance across and within districts.

The key findings over the 5 year study period (July 2018 to June 2023) were a decline in treatment interruptions, an increase in low-level viraemia (≥50 copies/ml), with AHD and high-level viraemia (≥1000 copies/ml) remaining stable. Whilst, stabilisation of virological non-suppession and high-level viraemia, is somewhat reassuring, the steady increase in low-level viraemia is worrying as persistent low level viraemia( > 50 to < 400) is a strong predictor of virological failure and low-level viraemia (400-999 copies/ml) is associated with increased risk of morbiditiy and mortality. Interestingly, during the study period the country experienced a pandemic as well as transitioning from a non-nucleoside reverse transcriptase inhibitor-based first line regimen to an integrase inhibitor-based regimen, both of which would be expected to influence the trends. A key strength of this study was the inclusion of a clearly defined inclusion criteria for the study outcomes, use of geospatial mapping and size of the study cohort.

The population level data presented is critical to closing the gaps to achieving the UNAIDS 95-95-95 target and addressing specific needs across geographic areas in a targeted manner. This well-written manuscript highlights the need for a more tailored and targeted approach to monitoring and closing the gaps and disparities in HIV care programmes across KwaZulu-Natal. And underscores the need for the inclusion of spatiotemporal monitoring for targeted and near real-time public health responses.

Minor revisions and comments:

A general comment, the authors may consider including the rationale for the selection of facilities and districts and not including all health districts in KwaZulu-Natal. Whilst this does not impact on the generalisability of the results, it certainly would contextualise the study for the reader.

Line 222-223: This sentence may need clarification: the interpretation of the reduced risk of viraemia in older clients, is it risk of viraemia (≥ 50copies/ml)? Authors need to be consistent with this term. While viraemia is defined as a detectable HIV viral load in plasma, further classification into low-level viraemia (≥ 50 - < 100 copies/ml) and high-level viraemia (≥ 1000 copies/ml) would improve interpretation of results and has programmatic implications. A further suggestion, the authors might consider classifying adjusted relative risk by HIV viral load thresholds (low-level versus high-level viraemia), this distinction will improve interpretation and application of these study findings. An increase in low-level viraemia(≥50 -<1000 copies/ml) is concerning as persistent HIV viral loads between 200 and 500 copies/ml is predictive of future virological failure and viral loads between 400 and 999 copies/ml are associated with increased 10-year risk of mortality. Distinguishing the two have implications for public health interventions.

Line 270- 271: Authors need to distinguish between low-level and high-level viraemia. General note, authors need to be consistent when classifying viraemia as “high-level viraemia” versus viraemia (≥1000 copies/ml) and “low-level viraemia” versus viraemia(≥ 50 copies/ml).

Line 290-292: Whilst the conclusion includes the key findings of the study and emphasises the need for more targeted programmatic interventions, the authors are encouraged to expand on the utility of geospatial analysis in a local context. Spatiotemporal analysis has the advantage of being used as near real-time monitoring and surveillance tool for informing targeted and tailored public health responses.

Reviewer #3: Trends in advanced HIV disease, treatment interruption, and viraemia in KwaZulu- Natal, South Africa: PGPH-D-25-03407

This manuscript makes a substantive contribution to HIV program monitoring by integrating longitudinal routine data with spatial visualization to analyze advanced HIV disease (AHD), treatment interruption, and viraemia. The study leverages a rich, programmatically relevant dataset (Tier.net) and addresses critical questions for hyper-endemic settings like KwaZulu-Natal. While the core regression methodology is strong, the spatial analysis component could be enhanced to move beyond descriptive visualization toward model-based inference.

Verdict: Major Revision

The abstract and intoduction sections are well written

Methods:

Statistical Analysis

1. Model Specification Issues:

a. Random Slope Specification: The phrase "random slope" is ambiguous. A random slope for which variable(s)? Typically, you would specify a random slope for a key predictor (e.g., year, or a clinic-level characteristic) if you hypothesize its effect varies by clinic. If not justified, an intercept-only model might be sufficient and more stable. Clarification is needed: Recommendation: Justify the choice of the variable(s) given random slopes. If exploring effect heterogeneity, consider model fit statistics (AIC, BIC) to compare with an intercept-only model.

b. Risk of Model Non-Convergence: Log-binomial models, especially with random effects, can suffer from convergence issues due to "non-collapsibility" and numerical instability.Recommendation: State how convergence was achieved (e.g., optimization algorithm, starting values). A robust alternative is a Poisson GLMM with a robust sandwich estimator or an over dispersed Poisson model, which is more stable and provides consistent estimates of RR, though confidence intervals may be conservative.

c. Temporal Confounding and Variable Timing: The description of client-level characteristics being summarized at three different time points (ART initiation, VL testing, first annual visit) is logical but complex. It risks introducing immortal time bias or confounding by time-updated factors if not handled meticulously. Recommendation: Provide a clear diagram or flowchart/table showing how covariates are aligned with each of the three analysis cohorts (AHD, viraemia, interruption). Consider explicitly framing these as separate, time-fixed analyses for distinct cohorts to avoid misinterpretation.

2. Variable Selection & Operationalization

a. Quintiles as Fixed Effects: Using quintiles (for clinic size, SAMPI) loses granular information and assumes a step-function relationship with the log-risk. The choice of quintiles should be justified.Recommendation: Consider using continuous variables with flexible specifications (e.g., splines) to model non-linear effects, or justify quintile categorization based on known thresholds or sample distribution.

b. "Year" as a Fixed Effect: Including year is good for temporal adjustment. However, specify its form (categorical vs. continuous). If categorical, state the reference year. If continuous, check the linearity assumption on the log-risk scale.

c. Missing Data: No mention of how missing data in covariates or outcomes is handled. Complete-case analysis can introduce bias.Recommendation: Report the amount of missingness for key variables and describe the method used (e.g., multiple imputation, which is preferred for mixed models).

3. Analysis Clarity & Reporting

a. Separate Models: It reads as if one giant model (or joint modeling for that matter) is fit for three distinct outcomes. This should be clarified.Recommendation: Explicitly state: "Separate models were fit for each outcome (AHD, treatment interruption, viraemia ≥50 copies/mL)."

b. Effect Measure Interpretation: The "clinic-level relative risk" phrasing could be misinterpreted. It's the RR associated with clinic-level characteristics (like size), not a RR per clinic.Recommendation: Rephrase to: "To estimate the association between clinic-level characteristics and the risk of [outcome], we expressed results as adjusted Relative Risks (aRR)..."

Geospatial mapping

1. Statistical and Methodological Weaknesses

a. Ignoring Uncertainty: This is the most critical flaw. IDW produces a single, smooth surface, giving a false impression of precision. It does not generate:

i. Prediction Intervals: How uncertain is the estimate at each unsampled location?

ii. Uncertainty Maps: Which areas have reliable estimates (near clinics) and which are highly speculative (far from clinics)?

iii. Policymakers might act on "hotspots" that are, in fact, statistical artifacts of low clinic density.

b. Naïve Assumption of Spatial Structure: IDW assumes a simple, deterministic distance-decay relationship. It does not model the underlying spatial process.

i. It ignores potential spatial autocorrelation beyond simple distance (e.g., directional trends, barriers like highways or rivers).

ii. It cannot separate signal (true spatial pattern) from noise (random clinic-level variation).

c. Sensitivity to Parameters: The IDW output is highly sensitive to the chosen power parameter (which controls the weight of distance) and search radius. The critique is incomplete without stating the values used and their justification.Recommendation: The authors must report the power parameter (e.g., power=2) and the search neighborhood strategy (e.g., number of nearest clinics used).

d. Edge Effects and Extrapolation: IDW performs poorly at the edges of the study area (e.g., near provincial borders) and can produce unrealistic "bullseye" artifacts around isolated clinics. It inappropriately extrapolates into areas with no data.

2. Inferential and Public Health Limitations

• Cannot Adjust for Covariates: The maps show crude proportions. A high proportion in a district could be due to its demographic (e.g., older population) or clinic-level (e.g., under-resourced) characteristics, not purely geography. This leads to ecological confounding. Recommendation: A geostatistical model like INLA can integrate the clinic- and client-level fixed effects from your GLMM directly into the spatial surface, producing an adjusted spatial risk map.

Results and discussion

Results and discussion sections could be improved with the improved approaches discussed above.

Reviewer #4: This is an important study to inform targeted interventions for the management of Advanced HIV Disease and Viremia across geographic locations. The authors note that data are unavailable due to legal and ethical requirements regarding clinical data in South Africa.

The following are the recommendations:

Abstract

Line 47-52: What was the total number of clients for the study? It is noted that 123,473 initiating ART had CD4 count tests done. May you also indicate the number of clients initiating ART who do not have their CD4 count tests? ( This will be helpful in addressing the gap of missed opportunities for AHD diagnosis and management). Also recommending an indication of clients who had their VL due but were not done.

Introduction

Line 71-75: May you also include a reference to the South African Guidelines regarding management of ART-AHD.

Line 93-95: Is there evidence for other provinces? Recommendation to include evidence for other provinces (from the 9 provinces, is the evidence only from Mpumalanga?)

Line 109-110: It is necessary to include the Viral load testing for viremia (management of viremia) as per the SA guidelines.

Results

Line 171-173: What was the total number of clients for the study? And disaggregation according to males and females

Line 177-178: Clarify the period for ART initiation (2018-2023) for the clients included in the AHD analysis.

Line 184-186: Figure 1- Flow chart for each study Outcome

A: Please add the number of clients with CD4>200 and CD4<200

C: Please add the number of clients with VL<50, VL>50-999 and VL>1000

These will help expand understanding of the burden of AHD, including viremia.

It would also be appreciated to disaggregate these outcomes by gender

Line 194-196: What were the findings for females?

Discussion

Line 243, 261, 274: What are the attributions for the geographical distributions (rural and urban) of AHD, Treatment Interruptions and Viremia? Also, what are the attributions for the gender disparities?

What were the observations regarding the management of viral load and viremia? (Line 211-212 showed that 76.9% of clients had VL results.) Also, the attributions for viremia in line 217 - geographical distribution.

**********

what does this mean?). If published, this will include your full peer review and any attached files.

**Do you want your identity to be public for this peer review?**  If you choose “no”, your identity will remain anonymous but your review may still be made public.

For information about this choice, including consent withdrawal, please see our Privacy Policy

Reviewer #1: **Yes:**  Kerusha Govender

Reviewer #2: No

Reviewer #3: **Yes:**  Elphas Okango

Reviewer #4: **Yes:**  Lufuno Malala

**********

[NOTE: If reviewer comments were submitted as an attachment file, they will be attached to this email and accessible via the submission site. Please log into your account, locate the manuscript record, and check for the action link "View Attachments". If this link does not appear, there are no attachment files.]

Attachment Submitted filename: PGPH-D-25-03407 Review.docx

# Author comment: Author response to Decision Letter 1

**DOI:** 10.1371/journal.pgph.0005506.r002

5 Jun 2026

Attachment Submitted filename: Response to Reviewers .docx

# Editor report: Decision Letter 1

**DOI:** 10.1371/journal.pgph.0005506.r003

**Authors:** Nei-yuan (Marvin) Hsiao

14 Aug 2026

Trends in advanced HIV disease, treatment interruption, and viraemia in KwaZulu-Natal, South Africa.

PGPH-D-25-03407R1

Dear Dr. Dorward,

We are pleased to inform you that your manuscript 'Trends in advanced HIV disease, treatment interruption, and viraemia in KwaZulu-Natal, South Africa.' has been provisionally accepted for publication in PLOS Global Public Health.

Before your manuscript can be formally accepted you will need to complete some formatting changes, which you will receive in a follow up email. A member of our team will be in touch with a set of requests.

Please note that your manuscript will not be scheduled for publication until you have made the required changes, so a swift response is appreciated.

IMPORTANT: The editorial review process is now complete. PLOS will only permit corrections to spelling, formatting or significant scientific errors from this point onwards. Requests for major changes, or any which affect the scientific understanding of your work, will cause delays to the publication date of your manuscript.

If your institution or institutions have a press office, please notify them about your upcoming paper to help maximize its impact. If they'll be preparing press materials, please inform our press team as soon as possible -- no later than 48 hours after receiving the formal acceptance. Your manuscript will remain under strict press embargo until 2 pm Eastern Time on the date of publication. For more information, please contact globalpubhealth@plos.org.

Thank you again for supporting Open Access publishing; we are looking forward to publishing your work in PLOS Global Public Health.

Best regards,

Nei-yuan (Marvin) Hsiao

Academic Editor

PLOS Global Public Health

***********************************************************

Reviewer Comments (if any, and for reference):
